# Orai1α, but not Orai1β, co-localizes with TRPC1 and is required for its plasma membrane location and activation in HeLa cells

**DOI:** 10.1007/s00018-021-04098-w

**Published:** 2022-01-06

**Authors:** Jose Sanchez-Collado, Jose J. Lopez, Isaac Jardin, Alejandro Berna-Erro, Pedro J. Camello, Carlos Cantonero, Tarik Smani, Gines M. Salido, Juan A. Rosado

**Affiliations:** 1 Department of Physiology (Cellular Physiology Research Group), Institute of Molecular Pathology Biomarkers (IMPB), University of Extremadura, 10003 Caceres, Spain; 2 Department of Physiology, (Smooth Muscle Physiology Research Group), Institute of Molecular Pathology Biomarkers, University of Extremadura, 10003 Caceres, Spain; 3 Department of Medical Physiology and Biophysics, University of Seville, Seville, Spain; 4 Group of Cardiovascular Pathophysiology, Institute of Biomedicine of Seville, University Hospital of Virgen del Rocio/University of Seville/CSIC, Seville, Spain

**Keywords:** Orai1α, Orai1β, STIM1, TRPC1, Store-operated calcium entry

## Abstract

The identification of two variants of the canonical pore-forming subunit of the Ca^2+^ release-activated Ca^2+^ (CRAC) channel Orai1, Orai1α and Orai1β, in mammalian cells arises the question whether they exhibit different functional characteristics. Orai1α and Orai1β differ in the N-terminal 63 amino acids, exclusive of Orai1α, and show different sensitivities to Ca^2+^-dependent inactivation, as well as distinct ability to form arachidonate-regulated channels. We have evaluated the role of both Orai1 variants in the activation of TRPC1 in HeLa cells. We found that Orai1α and Orai1β are required for the maintenance of regenerative Ca^2+^ oscillations, while TRPC1 plays a role in agonist-induced Ca^2+^ influx but is not essential for Ca^2+^ oscillations. Using APEX2 proximity labeling, co-immunoprecipitation and the fluorescence of G-GECO1.2 fused to Orai1α our results indicate that agonist stimulation and Ca^2+^ store depletion enhance Orai1α–TRPC1 interaction. Orai1α is essential for TRPC1 plasma membrane location and activation. Thus, TRPC1 function in HeLa cells depends on Ca^2+^ influx through Orai1α exclusively.

**Supplementary Information:**

The online version contains supplementary material available at 10.1007/s00018-021-04098-w.

## Introduction

Agonist-induced changes in cytosolic Ca^2+^ concentration are finely regulated to ensure the generation of spatiotemporally dynamic Ca^2+^ signals. The magnitude and pattern of Ca^2+^ signals depend on the type and concentration of the agonist; thus, low-intensity stimulation leads to oscillatory responses and higher-intensity stimulations generate more sustained responses [1, 2]. Store-operated Ca^2+^ entry (SOCE) is a mechanism for Ca^2+^ influx involved in a variety of cellular functions including gene transcription and the maintenance of Ca^2+^ oscillations [3–5]. SOCE is regulated by the filling state of the intracellular Ca^2+^ stores, primarily the endoplasmic reticulum (ER), so that occupation of phospholipase C-coupled plasma membrane agonist receptors leads to the generation of inositol trisphosphate (IP_3_), which, in turn, induces Ca^2+^ efflux from the intracellular Ca^2+^ stores. Store depletion triggers the activation of stromal interaction molecule (STIM) proteins leading to a conformational change that allows the interaction with and activation of Orai channels in the plasma membrane [6–10]. In addition to Orai1, two paralogs have been identified in mammalian cells, Orai2 and Orai3, which hetero-multimerize with Orai1 to fine-tune agonist-evoked Ca^2+^ responses [11]. In addition, two STIM isoforms have been described, STIM1 and STIM2. The Orai and STIM isoforms exhibit differential properties and are non-redundant tailoring-graded Ca^2+^ signals in response to physiological concentrations of agonists [11–14]. Two store-operated currents have been identified so far: the well-characterized and highly Ca^2+^-selective *I*_crac_, which involves activation of Orai channels by STIM proteins, and *I*_soc_, described in certain cell types, which involves the transient receptor potential (TRP) family member TRPC1, Orai1 and STIM1 [15, 16], that, unlike *I*_crac_, is non-selective for cations and exhibits higher conductance[17].

There is substantial evidence that TRPC1 channels are activated by multiple pathways, including receptor-operated (Ca^2+^ store-independent), store-operated (Orai1-dependent) and store-operated (Orai1-independent) mechanisms [18, 19]. This diversity is likely due to different heteromeric TRPC1 structures [20] and might be cell-specific, for instance, store-mediated activation of TRPC1 is Orai1-dependent in salivary glands [21], whereas a store-operated, Orai1-independent, TRPC1 activation has been described in vascular smooth muscle cells [22, 23], a pathway that coexists with a mechanism where TRPC1 functionally interacts with Orai1 and Ca_v_1.2 channels to mediate vasoconstriction [24].

A functional interaction between STIM1, Orai1 and TRPC1 has been reported in several cell types [21, 25]. It has been proposed that depletion of intracellular stores induces STIM1-dependent Ca^2+^ entry through Orai1 channels, thus leading to a local increase in free Ca^2+^ concentration that triggers the translocation of TRPC1 to the vicinity of STIM1 and Orai1 in the plasma membrane, where STIM1 can interact with and activate TRPC1 channels [16, 26]. A recent study using single-channel patch clamp experiments has revealed that endogenous TRPC1 forms a channel pore without involving Orai proteins [27] but its activation has been reported to be mostly dependent on Orai1-mediated Ca^2+^ influx. In this context, TRPC1 activation has been suggested to amplify and/or modify the pattern of Orai1-mediated Ca^2+^ signals [1].

Two Orai1 variants have been identified in mammalian cells, the long variant, named Orai1α, is the full-length Orai1 containing 301 amino acids, and the short variant, Orai1β arises from the same transcript by a process of alternative translation initiation from a methionine at position 64 in the Orai1α variant [28]. Some functional differences have been reported between both Orai1 forms, while both variants can support both *I*_crac_ and *I*_soc_ with similar efficiencies, only Orai1α is required for the formation of arachidonate-regulated channels underlying *I*_arc_. In addition, Orai1α is more sensitive to Ca^2+^-dependent inactivation [15], a mechanism that might involve specific phosphorylation of Orai1α at Ser34 in an adenylyl cyclase 8-dependent manner [4]. Using different experimental procedures, here we show that Orai1α functionally interacts with TRPC1 by a mechanism regulated by agonist stimulation. Functional Orai1α is essential both for the location of TRPC1 in the plasma membrane and for cation influx through the channel, by contrast, Orai1β, is not required for TRPC1 channel function or plasma membrane expression.

## Materials and methods

### Reagents and antibodies

Fura-2 acetoxymethyl ester (fura-2/AM) was from Molecular Probes (Leiden, The Netherlands). High-glucose Dulbecco’s modified Eagle’s medium (DMEM), fetal bovine serum, trypsin, penicillin/streptomycin, rabbit polyclonal anti-TRPC1 antibody (catalog number PA577303, epitope: amino acids 557–571 of human TRPC1), mouse monoclonal anti-PMCA antibody (clone 5F10; catalog number MA3-914, epitope: amino acids 724–783 of human PMCA), high-fidelity PCR kit (Platinum™ SuperFi™ DNA Polymerase), Clean-Blot™ IP detection reagent, SuperSignal^®^ West Dura extended duration substrate reagent, Pierce™ BCA protein assay kit, high-capacity streptavidin agarose resin and EZ-Link™ Sulfo-NHS-LC-Biotin were purchased from ThermoFisher Scientific (Waltham, MA, USA). Complete EDTA-free protease inhibitor cocktail tablets were from Roche Diagnostics GmbH (Mannheim, Germany). DharmaFECT kb transfection reagent was obtained from Horizon Discovery (Waterbeach, UK). *N*-Glycosidase F (PNGase F) was purchased from New England Biolabs Inc (Ipswich, MA, USA). Mouse monoclonal Anti-GOK/Stim1 antibody (Clone 44/GOK; catalog number 610954, epitope: amino acids: 25–139 of human STIM1) was purchased from BD Biosciences (San Jose, CA, USA). Thapsigargin (TG), histamine, protein A agarose beads, HEPES (4-(2-Hydroxyethyl)piperazine-1-ethanesulfonic acid), EGTA (ethylene glycol-bis(2-aminoethylether)-*N*,*N*,*N*′,*N*′-tetraacetic acid), EDTA (ethylenedinitrilotetraacetic acid), bovine serum albumin (BSA), biotin fenol, trolox, sodium azide, sodium ascorbate, biotin, rabbit polyclonal anti-Orai1 antibody (catalog number O8264, epitope: amino acids 288–301 of human Orai1), rabbit polyclonal anti-Orai1 (AB-1) antibody (catalog number AV50117, epitope: amino acids 2–61 of human Orai1) and rabbit polyclonal anti-β-actin antibody (catalog number A2066, epitope: amino acids 365–375 of human β-actin) were obtained from Sigma (St Louis, MO, USA). Horseradish peroxidase-conjugated goat anti-mouse immunoglobulin G (IgG) antibody and goat anti-rabbit IgG antibody were from Jackson laboratories (West Grove, PA, USA). CMV-promoter EYFP-Orai1 plasmid (which might give Orai1α and Orai1β) was kindly provided by Christoph Romanin (Institute of Biophysics, Johannes Kepler University Linz, Austria). CMV-promoter Orai1α-EGFP and Orai1β-EGFP plasmids were kindly provided by Mohamed Trebak (Department of Cellular and Molecular Physiology, The Pennsylvania State University, Hershey, PA, USA), been optimized to give essentially 100% Orai1α and 100% Orai1β, respectively. Green fluorescent genetically encoded Ca^2+^ indicator for optical imaging (version 1.2) (G-GECO1.2)-Orai1 was a gift from Michael Cahalan (Addgene plasmid #73562; http://n2t.net/addgene:73562; Research Resource Identifier: Addgene_73562). TRPC1-flag was a gift from Craig Montell (Addgene plasmid # 24408; http://n2t.net/addgene:24408; RRID:Addgene_24408). pRK5-HA-TRPC1-F562A (dnTRPC1) plasmid was a gift from Jessica Sabourin (Inserm, UMR-S 1180, Signalisation et Physiopathologie Cardiovasculaire, Université Paris-Saclay, Châtenay-Malabry, France). MO7O-Orai1-E106Q-flag (dnOrai1) was provided by Thierry Capiod (INSERM U1151, Institut Necker Enfants Malades, Université Paris Descartes, Paris, France). MO91-STIM1-CFP was a gift from Anjana Rao (Addgene plasmid #19755; http://n2t.net/addgene:19755; RRID:Addgene_19755). All other reagents were of an analytical grade.

### Site-directed mutagenesis

Plasmids encoding GECO-Orai1 and Orai1β-EGFP were used as template to generate their respective dominant-negative mutants GECO-Orai1-E106Q and Orai1βE43Q-EGFP (corresponding to the Orai1αE106Q-EGFP mutant), using high-fidelity PCR kit (Platinum™ SuperFi™ DNA Polymerase, Invitrogen) and the following primers; ORAI1-E106Q_F: 5′-GGTGGCAATGGTGCAGGTGCAGCTGGA-3′, ORAI1-E106Q_R: 5′-TCCAGCTGCACCTGCACCATTGCCACC-3′. Sequencing analysis results are depicted in Fig. S1.

### Cell culture and transfections

HeLa cells were obtained from the American Type Culture Collection (CCL-2; Mansassas, VA, USA) and cultured at 37 °C with a 5% CO_2_ in high-glucose DMEM supplemented with 10% (v/v) fetal bovine serum and 100 U/mL penicillin and streptomycin, as described previously [29]. For transient transfections, cells were grown to 60–80% confluency and transfected with expression plasmids for Orai1α-GFP, Orai1-β-GFP, dn-Orai1α, dnOrai1β-GFP, GECO-Orai1, GECO-dnOrai1 and STIM1-CFP, TRPC1-FLAG and HA-TRPC1-F562A, depending on the experimental conditions, using DharmaFECT kb transfection reagent and were used 48 h after transfection. For a 4-component overexpression system (STIM1, Orai1α, Orai1β, TRPC1), we use a DNA ratio of 4:1:1:2, and for the 3-component overexpression system (STIM1, GECO-Orai1α, TRPC1 or STIM1, Orai1α/Orai1β, TRPC1), we use a DNA ratio of 2:1:1. For Western blotting, immunoprecipitation and biotinylation assays, cells (2 × 10^6^) were plated in 100-mm Petri dish and cultured for 48 h, while, for calcium imaging, determination of Mn^2+^ entry and confocal determination of G-GECO1.2 fluorescence assays, cells (4 × 10^5^) were seeded in a 35-mm six-well multi-dish.

### Immunoprecipitation and Western blotting

Immunoprecipitation and Western blotting were performed as described previously [30]. Briefly, cells cultured on 100-mm Petri dish (8 × 10^6^ cells) were stimulated with 3 μM histamine or with vehicle and subsequently lysed with ice-cold NP-40 buffer pH 8 containing 137 mM of NaCl, 20 mM of Tris, 2 mM of EDTA, 10% glycerol, 1% nonidet P-40, 1 mM of Na_3_VO_4_, and complete EDTA-free protease inhibitor tablets. Cell lysates (1 mL) were immuno-precipitated by incubation with 2 µg of anti-Orai1, anti-STIM1 or anti-TRPC1 antibody and 50 µL of protein A-agarose overnight at 4 °C on a rotary platform. Orai1 variants expression and detection were analyzed after protein de-glycosilation by treatment of whole-cell lysates with PNGase F according to the protocols provided by the manufacturer. Cell lysates and immuno-precipitates were resolved by 10% or 12% SDS-PAGE and separated proteins were electrophoretically transferred onto nitrocellulose membranes for subsequent probing. Blots were incubated overnight with 10% (w/v) BSA in Tris-buffered saline with 0.1% Tween 20 (TBST) to block residual protein-binding sites. Immuno-detection of Orai1 variants, β-actin, STIM1, PMCA and TRPC1 was achieved by incubation for 1 h with anti-Orai1 antibody diluted 1:1000 in TBST, 1 h with anti-β-actin antibody diluted 1:2000 in TBST, 1 h with anti-STIM1 diluted 1:500 in TBST, 2 h with anti-PMCA diluted 1:1000 in TBST or 2 h with anti-TRPC1 diluted 1:1000 in TSBT. The primary antibody was removed, and blots were washed six times for 5 min each with TBST. To detect the primary antibody, blots were incubated for 1 h with horseradish peroxidase-conjugated goat anti-mouse IgG antibody, horseradish peroxidase-conjugated goat anti-rabbit IgG antibody diluted 1:10,000 in TBST, or Clean-Blot™ IP Detection Reagent diluted 1:250 in TBST, and then exposed to enhanced chemiluminescence reagents for 5 min. The antibody binding was assessed with a C-DiGit Chemiluminescent Western Blot Scanner (LI-COR Biosciences, Lincoln, NE, USA) and the density of bands was measured using ImageJ software v.1.8.0_172 (NIH, Bethesda, MD, USA). Data were normalized to the amount of protein recovered by the antibody used for the immunoprecipitation or to β-actin from the same gel.

### APEX2 proximity labeling assay

The APEX2 proximity labeling assays were performed as described previously [31]. Briefly, biotin–phenol labeling was initiated by cell incubation at 37 °C with a 5% CO_2_ for 30 min in cell culture medium supplemented with 2.5 mM biotin–phenol. Cells were stimulated with 3 μM histamine for 5 min or with vehicle, then medium was removed and cells were washed three time with PBS (10 mM Na_2_HPO_4_, 1.8 mM KH_2_PO_4_, 2.7 mM KCL, 140 mM NaCL, 0.5 mM MgCl_2_, 1 mM CaCl_2,_ pH 7.4) and incubated for 1 min in biotinylation buffer (PBS supplemented with 1 mM H_2_O_2_). Later, biotinylation buffer was aspired and cells were washed three times with STOP/wash buffer (PBS containing: 0.5 mM MgCl_2_, 1 mM CaCl_2_, 5 mM Trolox, 10 mM sodium ascorbate, 10 mM sodium azide). Cells were subsequently lysed with ice-cold lysis (RIPA) buffer pH 8 (50 mM Tris, 150 mM NaCl, 5 mM EDTA, 0.5% sodium deoxycholate, 0,1% SDS, 1% Triton X-100, complete EDTA-free protease inhibitor cocktail) supplemented with 5 mM Trolox, 10 mM sodium ascorbate, 10 mM sodium azide and then cells were scraped. Cell lysates were transferred to micro-centrifuge tubes and samples were sonicated. Clear cell lysates were obtained by centrifugation at 20,000 × *g* for 30 min at 4 °C and total protein concentrations were determined using the BCA method. Biotinylated proteins were isolated by incubation with 50 µL of streptavidin beads at 4 °C for 2 h on a rotary platform. Later, beads were washed twice with 1 mL RIPA buffer, once with 1 mL 1 M KCl, once with 1 mL 0.1 M Na_2_CO_3,_ once with 1 mL 2 M urea in Tris–HCl pH 8.0 and once with 1 mL RIPA buffer. For Western blotting assay, the biotinylated and non-biotinylated fractions were then eluted by boiling the beads at 95 °C for 15 min in Laemmli buffer (0.62 M Tris–Cl pH 6.8, 2% SDS, 10% glycerol, 0.002% bromophenol blue) supplemented with 100 mM DTT and 1 mM biotin, and Western blotting was performed as described above.

### Determination of cytosolic free Ca^2+^ concentration

Cells were loaded with fura-2 by incubation with 5 μM fura-2/AM for 30 min at 37 °C. Coverslips with cultured cells were mounted on a perfusion chamber and placed on the stage of an epifluorescence inverted microscope (Nikon Eclipse Ti2, Amsterdam, The Netherlands) with an image acquisition and analysis system for video-microscopy (NIS-Elements Imaging Software v.5.02.00, Nikon, Amsterdam, The Netherlands). Cells were continuously super-fused at room temperature with HEPES-buffered saline (HBS) containing (in mM) 125 NaCl, 5 KCl, 1 MgCl_2_, 5 glucose, and 25 HEPES, pH 7.4, supplemented with 0.1% (*w*/*v*) BSA. Cells were examined at 40× magnification (Nikon CFI S FLUOR 40× Oil, Amsterdam, The Netherlands) and were alternatively excited with light from a xenon lamp passed through a high-speed monochromator Optoscan ELE 450 (Cairn Research, Faversham, UK) at 340/380 nm. Fluorescence emission at 505 nm was detected using a cooled digital sCMOS camera Zyla 4.2 (Andor, Belfast, UK) and recorded using NIS-Elements AR software (Nikon, Amsterdam, The Netherlands). Fluorescence ratio (F340/F380) was calculated pixel by pixel, and the data were presented as ΔF_340_/F_380_. Histamine-evoked changes in cytosolic free Ca^2+^ concentration were estimated as the area under the curve measured as the integral of the rise in fura-2 fluorescence ratio 10 min after the addition of histamine and taking a sample every second. TG-induced Ca^2+^ entry was estimated as the integral of the rise in fura-2 fluorescence ratio 2.5 min after the addition of Ca^2+^ and taking a sample every second.

### Analysis of Ca^2+^ oscillations

The analysis of Ca^2+^ oscillations was performed as described previously [13]. All Ca^2+^ traces obtained in the Ca^2+^-imaging experiments were plotted using GraphPad Prism v.8.4.3 (GraphPad Software, San Diego, CA, USA). The numbers of oscillations per 10 min were manually counted. Then, cells were classified in three groups and a percentage of each group of cells was calculated for each individual coverslip. The first group, oscillating cells, includes cells showing regenerative oscillations after histamine stimulation for the duration of the experiment, where each oscillation returns to baseline before the start of the next oscillation. A second group, plateau cells, includes cells showing a sustained a cytosolic Ca^2+^ signal that was ≥ 25% of the initial peak, for at least 5 min after stimulation. Finally, the last group includes cells that show either no response to histamine stimulation or showed only one initial spike and remained at baseline for the duration of the recording. All manual counts were independently recorded by two individuals to ensure accuracy of counts.

### Determination of Mn^2+^ entry

Cells were loaded with fura-2 by incubation with 5 μM fura-2/AM for 30 min at 37 °C. Coverslips with cultured cells were mounted on a perfusion chamber and placed on the stage of an epifluorescence inverted microscope (Nikon Eclipse Ti2, Amsterdam, The Netherlands) with an image acquisition and analysis system for video-microscopy (NIS-Elements Imaging Software v.5.02.00, Nikon, Amsterdam, The Netherlands). Cells were continuously super-fused at room temperature with HEPES-buffered saline (HBS) containing (in mM) 1 CaCl_2_, 0.5 MnCl_2_, 125 NaCl, 5 KCl, 1 MgCl_2_, 5 glucose, and 25 HEPES, pH 7.4, supplemented with 0.1% (*w*/*v*) BSA. Cells were examined at 40× magnification (Nikon CFI S FLUOR 40× Oil, Amsterdam, The Netherlands) and were excited with light from a xenon lamp passed through a high-speed monochromator Optoscan ELE 450 (Cairn Research, Faversham, UK) at 360 nm. Fluorescence emission at 505 nm was detected using a cooled digital sCMOS camera Zyla 4.2 (Andor, Belfast, UK) and recorded using NIS-Elements AR software (Nikon, Amsterdam, The Netherlands). Mn^2+^ influx was monitored as the quenching of fura-2 fluorescence at the isoemissive wavelength of 360 nm and presented on an arbitrary linear scale. To compare the rate of decay of fura-2 fluorescence when cells were subjected to different experimental procedures, traces were fitted to the equation *y* = *S* × *e*^−*KX*^ + *A*, where *K* is the slope, *S* is the span and *A* is the plateau, as described previously [32].

### Confocal determination of G-GECO1.2 fluorescence

G-GECO1.2-Orai1 or G-GECO1.2-dnOrai1-transfected Hela cells were seeded on coverslips and mounted on a perfusion chamber and placed on the stage of an epifluorescence inverted microscope (Nikon Eclipse Ti, Amsterdam, The Netherlands) with an image acquisition and analysis system for video-microscopy NIS-Elements Imaging Software v.5.02.00, (Nikon, Amsterdam, The Netherlands). Cells were continuously super-fused with HBS supplemented with 0.1% (*w*/*v*) BSA at room temperature. Cells were examined at 60× magnification and excited using a confocal laser-scanning system (Melles-Griot, IDEX Health & Science, Wallingford, CT, USA) at 488 nm. Fluorescence emission at 515 nm was detected and recorded using NIS-Elements AR software (Nikon, Amsterdam, The Netherlands). GECO fluorescence was determined before and after the addition of 3 µM histamine (resting) in presence of extracellular Ca^2+^ (1 mM) for 10 min. Images were analyzed using ImageJ software v.1.8.0_172 (NIH, Bethesda, MD, USA).

### Biotinylation of cell surface proteins

The labeling and isolation of cell surface proteins were performed by surface biotinylation assay, as described previously [33]. Hela cells grown in 75 cm^2^ tissue culture dishes were washed three times with phosphate-buffered saline (PBS, NaCl 137 mM, KCl 2.7 mM, KH_2_PO4, 1.5 mM, Na_2_HPO_4_·2H_2_O 8 mM, pH 8). Later, cells were incubated at 4 °C for 1 h with biotynilation buffer (PBS supplemented with 1 mg/mL EZ-Link sulfo-NHS-LC-biotin) The biotinylation reaction was terminated by addition of Tris base to a final concentration of 50 mM. Following biotinylation, cells were washed twice in PBS, disrupted using Nonidet P-40 buffer and sonicated. Cell lysates were centrifuged (16,000×*g* for 5 min at 4 °C) and protein concentration was measured using BCA assay. Samples were incubated with 50 µL streptavidin beads at 4 °C for 2 h and re-suspended in Laemmli buffer for subsequent analysis by Western blotting. The biotinylated and non-biotinylated fractions were separated in 8% SDS-PAGE, TRPC1 surface expression was detected using a specific anti-TRPC1 antibody, while the detection of PMCA was used as control.

### Statistical analysis

All data are presented as the mean ± standard error of mean (SEM). Analysis of statistical significance was performed using GraphPad Prism v.8.4.3 (GraphPad Software, San Diego, CA, USA). Kruskal–Wallis test combined with Dunn´s post hoc test (or one-way analysis of variance combined with Tukey post hoc test for the analysis of Ca^2+^ determinations) was used to compare the different experimental groups. For comparison between two groups, the Mann–Whitney U test was used. Throughout the manuscript *, **, and *** indicate *p* values of < 0.05, < 0.01, and < 0.001, respectively. All data with *p* < 0.05 were deemed significant; “ns” = nonsignificant.

## Results

### Orai1α and Orai1β, but not TRPC1, are important to modulate Ca^2+^ oscillations

**Fig. 1:**
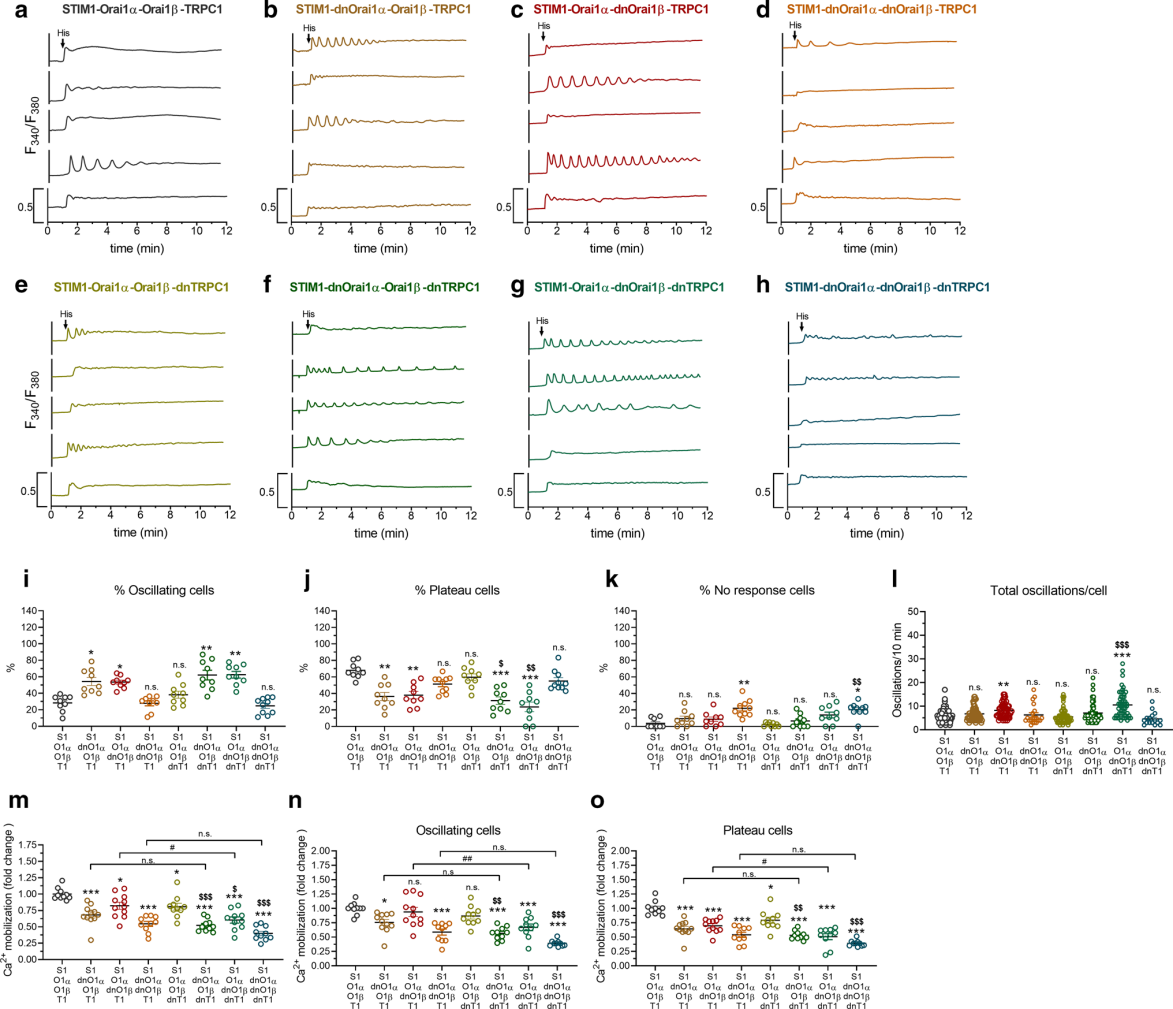
Orai1α and Orai1β, but not TRPC1, are important to modulate Ca^2+^ oscillations. **a**–**h** Representative Ca^2+^ oscillations in response to 3 µM histamine measured using fura-2 in HeLa cells co-transfected with STIM1, Orai1α, Orai1β and TRPC1 or the corresponding dominant-negative mutants, as described. Cells were super-fused with HBSS containing 1 mM Ca^2+^ and stimulated with 3 µM histamine at 1 min (indicated by arrow). Representative traces from five cells/condition were chosen to represent the datasets. **i**–**l** Quantification of the percentage of oscillating cells (**i**), percentage of plateau cells (**j**), percentage of non-responding cells (**k**) and total oscillations/cell in 10 min (**l**) for data presented in **a**–**h** (for **i** to **k**, *n* = 9; *n* values correspond to independent experiments; for **l**, from left to right, *n* = 99, 68, 46, 20, 73, 62, 53 and 16; n values correspond to individual cells). **m**–**o** Quantification of Ca^2+^ mobilization for all the conditions from a to h estimated in all the cells (**m**), oscillating cells (**n**) and plateau cells (**o**). Scatter plots are represented as mean ± SEM and were statistically analyzed using Kruskal–Wallis test with multiple comparisons (Dunn’s test) to HeLa cells expressing WT Orai1α, Orai1β and TRPC1 (*p < 0.05, **p < 0.01, and ***p < 0.001), HeLa cells expressing WT Orai1α, WT Orai1β and dnTRPC1 (for conditions including the expression of dnTRPC1; ^$^*p* < 0.05, ^$$^*p* < 0.01, and ^$$$^*p* < 0.001) or the corresponding condition with WT TRPC1 vs dnTRPC1 (^#^*p* < 0.05, ^##^*p* < 0.01, and ^###^*p* < 0.001).

We have analyzed the functional role of Orai1α, Orai1β and TRPC1 on histamine-induced Ca^2+^ oscillations. HeLa cells were co-transfected with STIM1-CFP, Orai1α-EGFP, Orai1β-EGFP and TRPC1 or the corresponding dominant-negative mutants for Orai1α (Orai1α E106Q), Orai1β (Orai1βE43Q-EGFP corresponding to the E106Q mutant of the long Orai1 variant) and TRPC1 (HA-TRPC1-F562A). Expression of the different plasmids was demonstrated by Western blotting using the appropriate antibodies (Fig. S2). Cells were stimulated with 3 µM histamine to elicit Ca^2+^ oscillations in the presence of 1 mM extracellular Ca^2+^. Traces from five representative cells are shown in Fig. 1a–h. An average of 29% of HeLa cells expressing WT STIM1, Orai1α, Orai1β and TRPC1 stimulated with histamine responded with regenerative Ca^2+^ oscillations (Fig. 1i), with an average of 5.7 ± 0.2 oscillations/10 min (Fig. 1l). Of the cells that did not oscillate, 68% responded with a sustained plateau and the remaining 3% of cells did not respond (Fig. 1j, k). In mock-treated cells, an average of about 20% responded with Ca^2+^ oscillations after stimulation with histamine, with an average of 4 oscillations/10 min (Fig. S3a–c). As expected, the magnitude of Ca^2+^ mobilization upon treatment with histamine was significantly greater in cells expressing STIM1, Orai1α, Orai1β and TRPC1 (Fig. S3d; *p* < 0.001).

When Orai1α or Orai1β were substituted by Orai1α-E106Q (dnOrai1α) or Orai1β-E43Q (dnOrai1β) mutants, the percentage of cells that responded with Ca^2+^ oscillations significantly increased (57% and 56%, for Orai1α and Orai1β, respectively; *p* < 0.05; Fig. 1b, c, i). Interestingly, expression of the dnOrai1β mutant significantly enhanced the average number of oscillations per cell (8.2 ± 0.4 oscillations/10 min; Fig. 1l). However, co-transfection of HeLa cells with STIM1, TRPC1 and both dominant-negative mutants of Orai1α and Orai1β returned the percentage of cells that responded with Ca^2+^ oscillations to that observed in cells expressing WT STIM1, Orai1α, Orai1β and TRPC1 (Fig. 1d, i).

To ascertain the possible role TRPC1 in the maintenance or Ca^2+^ oscillations, we repeated the previously described protocols in the presence of the dominant-negative TRPC1 mutant (HA-TRPC1-F562A; dnTRPC1). As shown in Fig. 1e–l, substitution of WT TRPC1 for dnTRPC1 did not significantly alter the response pattern of the cells to histamine. Thus, suggesting that TRPC1 does not play a relevant role in the maintenance of regenerative Ca^2+^ oscillations in these cells.

Concerning the magnitude of histamine-evoked Ca^2+^ mobilization, co-transfection of dnOrai1α or dnOrai1β, instead of the WT Orai1 variants, with STIM1 and TRPC1 led to a significant attenuation of histamine-evoked Ca^2+^ mobilization (Fig. 1m; *p* < 0.05), which affected exclusively to the cells that responded with a sustained plateau (Fig. 1n, o). Consistent with this, substitution of both WT Orai1 variants for their respective dominant-negative mutants further decreased Ca^2+^ mobilization induced by histamine, which, in this case, affected to both oscillating cells and cells that responded with a sustained plateau (Fig. 1m–o; *p* < 0.001). Co-transfection of dnTRPC1 with STIM1, Orai1α and Orai1β (or the corresponding dnOrai1α and dnOrai1β) significantly decreased histamine-evoked Ca^2+^ mobilization as compared to HeLa cells expressing WT TRPC1 (Fig. 1m–o; *p* < 0.05 to 0.001). In the presence of dnTRPC1, agonist-induced Ca^2+^ mobilization was still smaller when dnOrai1α, dnOrai1β or both were expressed (Fig. 1n, o). These findings indicate that, while Orai1α, Orai1β and TRPC1 are required for histamine-induced Ca^2+^ mobilization, only Orai1α and Orai1β play a relevant role in the maintenance of regenerative Ca^2+^ oscillations.

We have further analyzed the functional role of Orai1α, Orai1β and TRPC1 on histamine-induced Ca^2+^ oscillations using triple co-expression of STIM1, Orai1α or Orai1β and TRPC1, or the corresponding dominant-negative mutants. Traces from five representative cells are shown in Fig. S4a–h. With this experimental maneuver, an average of 51 ± 3 and 52 ± 5% of HeLa cells expressing WT STIM1, Orai1α or Orai1β, respectively, and TRPC1 stimulated with histamine responded with regenerative Ca^2+^ oscillations (Fig. S4i), with an average of 9.2 ± 0.7 and 7.6 ± 0.7 oscillations/10 min, for Orai1α and Orai1β (Fig. S4l). When the WT Orai1 variants were replaced by their corresponding dominant-negative mutants, the percentage of cells that responded with Ca^2+^ oscillations significantly decreased to 18 ± 3% and 21 ± 2%, for Orai1α and Orai1β, respectively, and the average number of oscillations per cell was attenuated (3.8 ± 0.6 and 4.6 ± 0.5 oscillations/10 min, for Orai1α and Orai1β, respectively; Fig. S4c, d, i, l; *p* < 0.05). As expected, substitution of the Orai1 variants by their respective dominant-negative mutants significantly attenuated the magnitude of histamine-evoked Ca^2+^ mobilization (Fig. S4m–o; *p* < 0.05). These findings confirm that both variants of Orai1 are required for agonist-induce Ca^2+^ oscillations. We have further evaluated the role of TRPC1 in this triple co-expression system replacing TRPC1 by dnTRPC1. Substitution of WT TRPC1 for dnTRPC1 did not significantly alter the response pattern of the cells to histamine as reported in Fig. 1, thus suggesting that TRPC1 does not play a relevant role in the maintenance of regenerative Ca^2+^ oscillations in these cells. Interestingly, expression of dnTRPC1 instead of WT TRPC1 significantly attenuated the magnitude of agonist-evoked Ca^2+^ mobilization exclusively in cells expressing functional Orai1α (Fig. S4m; *p* < 0.01).

### Analysis of the interaction of Orai1α and Orai1β with TRPC1

**Fig. 2:**
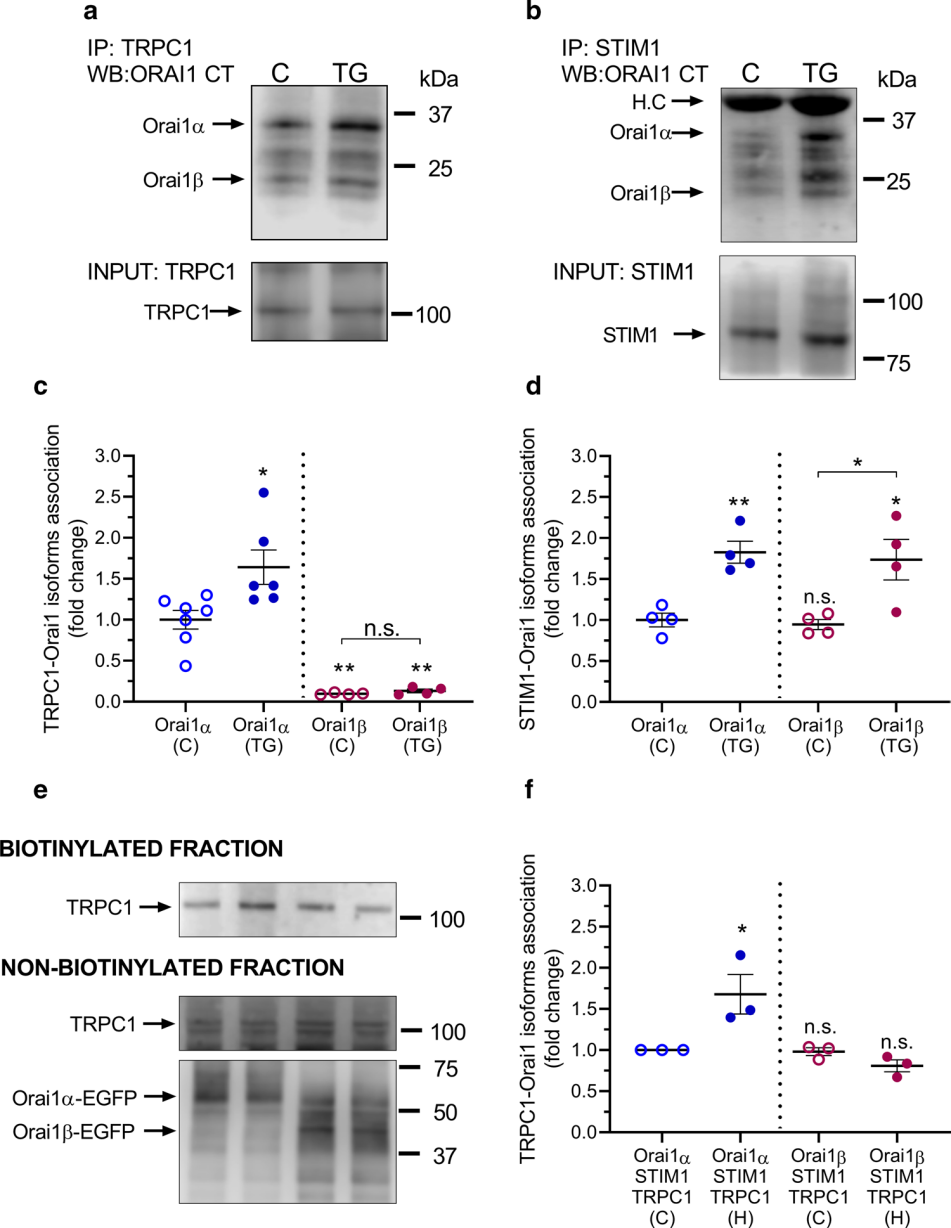
Interaction of Orai1α and Orai1β with TRPC1 and STIM1. HeLa cells were suspended in HBS containing 1 mM Ca^2+^ and then stimulated for 1 min with 2 µM TG (TG) or the vehicle (C) and lysed. Whole-cell lysates were immuno-precipitated with anti-TRPC1 (**a**) or anti-STIM1 antibody (**b**). Immuno-precipitates were treated with PNGaseF and then subjected to 10% SDS-PAGE and Western blotting with the anti-Orai1 antibody, as described in Material and Methods. Membranes were re-probed with the antibody used for immunoprecipitation for protein loading control. Molecular masses indicated on the right were determined using molecular-mass markers run in the same gel. Blots are representative of four to seven separate experiments. **c**, **d** Scatter plots, representing the quantification of the TRPC1-Orai1α/β or STIM1-Orai1α/β interaction, are presented as mean ± SEM and were statistically analyzed using the Mann–Whitney U test (*p < 0.05 and **p < 0.01, as compared to the corresponding control (C)). **e** HeLa cells were co-transfected with STIM1-CFP, TRPC1 and either Orai1α-GFP (lanes 1 and 2) or Orai1β-GFP (lanes 3 and 4). Cells were suspended in HBS containing 1 mM Ca^2+^ and then stimulated with 3 µM histamine (lanes 2 and 4) or the vehicle (lanes 1 and 3) for 5 min, and the interaction between Orai1 variants and TRPC1 was assessed by APEX2 proximity labeling assay, as described in Material and Methods. The biotinylated and non-biotinylated fractions were subjected to 10% SDS-PAGE and Western blotting with the anti-TRPC1 antibody. The non-biotinylated fraction was also probed with the anti-Orai1antibody for protein loading control. Molecular masses indicated on the right were determined using molecular-mass markers run in the same gel. Blots are representative of three separate experiments. **f** Scatter plots, representing the quantification of the TRPC1-Orai1α or TRPC1-Orai1β interaction in resting (C; control) and histamine (H)-treated cells, are presented as mean ± SEM and were statistically analyzed using the Mann–Whitney *U* test (*p < 0.05, as compared to the corresponding control). *HC* heavy chain of the IgG used for immunoprecipitation

We have explored the interaction between the Orai1 variants, Orai1α and Orai1β, and TRPC1 in native HeLa cells by looking for co-immunoprecipitation from cell lysates. Immunoprecipitation and subsequent SDS/PAGE and Western blotting were conducted using resting cells and cells in which the intracellular Ca^2+^ stores had been depleted by 1 min treatment with thapsigargin (TG; 2 µM) in the presence of extracellular Ca^2+^ (1 mM). After immunoprecipitation with the anti-TRPC1 antibody, and protein de-glycosylation with PNGaseF, Western blotting revealed the presence of Orai1α and, in a significantly less amount, Orai1β in samples from resting cells. (Fig. 2a, top panel). Treatment with TG significantly increased the amount of Orai1α immuno-precipitated with anti-TRPC1 antibody (Fig. 2a, top panel, and c; *p* < 0.05) without having any effect on the interaction between Orai1β and TRPC1 (Fig. 2a, c). Western blotting with anti-TRPC1 antibody confirmed a similar amount of protein in all lanes (Fig. 2a, bottom panel).

Previous studies have demonstrated functional interaction of both Orai1 variants with STIM1 [4, 15]. As a positive control of our experimental procedure, we have evaluated the interaction of STIM1 with Orai1α and Orai1β following the previously described experimental maneuver. As shown in Fig. 2b, top panel, after immunoprecipitation with the anti-STIM1 antibody and protein de-glycosylation with PNGaseF, Western blotting reveals a low amount of Orai1 variants associated with STIM1 in resting cells. The association of Orai1α and Orai1β with STIM1 significantly increased after treatment with TG (Fig. 2b, d; *p* < 0.05). Western blotting with anti-STIM1 antibody confirmed a similar amount of protein in all lanes (Fig. 2b, bottom panel).

We also analyzed the interaction between TRPC1 and Orai1 variants by immunoprecipitation using an alternative experimental maneuver. As shown in Fig. S5, HeLa cells were suspended in a Ca^2+^-free medium (300 µM EGTA added) or a medium containing 1 mM CaCl_2_ and were stimulated with TG or left untreated and lysed. Orai1α was pulled down by immunoprecipitation of the whole-cell lysates with anti-Orai1 N-terminal antibody (epitope: amino acids 2–61 exclusive to Orai1α). The immuno-precipitates were subjected to SDS/PAGE and Western blotting with anti-TRPC1 antibody to detect the Orai1α-TRPC1 association. On the other hand, Orai1β was pulled down from the supernatant by immunoprecipitation with anti-Orai1 (C-terminal) antibody (epitope: amino acids 288–301 present in both Orai1 variants). The resulting pellet was subjected to SDS/PAGE and Western blotting with anti-TRPC1 antibody to detect the Orai1β-TRPC1 association. A detectable association was found between Orai1α and TRPC1 both in the absence and presence of extracellular Ca^2+^ but we were unable to detect a significant interaction between TRPC1 and Orai1β (Fig. S5).

We have further tested the interaction between Orai1 variants and TRPC1 by proximity-dependent biotinylation using APEX2. HeLa cells were co-transfected with STIM1-CFP, TRPC1 and either Orai1α-EGFP or Orai1β-EGFP. Experiments were performed in resting cells and cells stimulated with 3 µM histamine for 5 min in the presence of extracellular Ca^2+^ (1 mM). As shown in Fig. 2e, top panel, the resulting biotinylation pattern reveals a small but detectable interaction of TRPC1 with Orai1α and Orai1β in resting conditions. Interestingly, treatment with histamine significantly enhances the Orai1α–TRPC1 interaction without inducing any modification in the interaction between TRPC1 and Orai1β (Fig. 2e, top panel, and f). Western blot analysis of the non-biotinylated fraction with anti-TRPC1 antibody revealed the amount of TRPC1 not associated to Orai1 variants (Fig. 1e, middle panel). Further, re-probing of the non-biotinylated fraction with anti-Orai1 (C-terminal) antibody confirmed a similar amount of Orai1 variants in all lanes (Fig. 2e, bottom panel).

Altogether, either using co-immunoprecipitation or APEX2 proximity labeling assay, our results indicate that there is a detectable interaction of TRPC1 with Orai1α under resting conditions that was significantly enhanced by treatment with TG or physiological agonists. By contrast, there is a small, if any, interaction between Orai1β and TRPC1 that seems constitutive in nature.

**Fig. 3:**
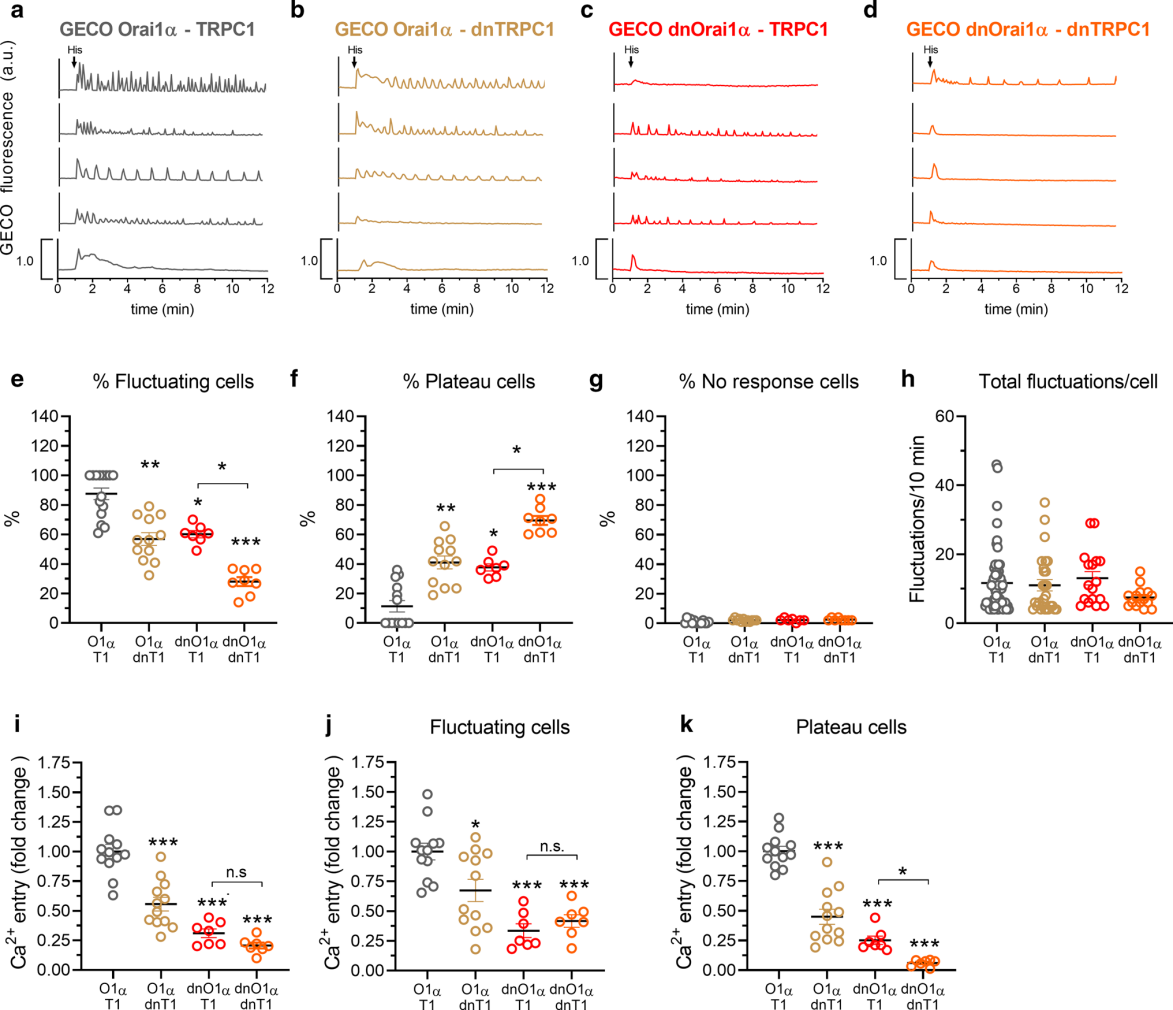
TRPC1 modulates the function of Orai1α. **a**–**d** Representative Ca^2+^ responses to 3 µM histamine measured using G-GECO1.2 in HeLa cells co-transfected with STIM1, G-GECO1.2-Orai1α and TRPC1 or the corresponding dominant-negative mutants, as described. Cells were super-fused with HBSS containing 1 mM Ca^2+^ and stimulated with 3 µM histamine at 1 min (indicated by arrow in **a**). Representative traces from five cells/condition were chosen to represent the datasets. **e**–**h** Quantification of the percentage of fluctuating cells (**e**), percentage of plateau cells (**f**), percentage of non-responding cells (**g**) and total fluctuations/cell in 10 min (**h**) for data presented in **a**–**d** (for **e**–**g**, *n* = 7–14; *n* values correspond to independent experiments; for h, from left to right, *n* = 65, 27, 17 and 15; *n* values correspond to individual cells). **i**–**k** Quantification of Ca^2+^ entry for all the conditions from a to d estimated in all the cells (**i**), fluctuating cells (**j**) and plateau cells (**k**). Scatter plots are represented as mean ± SEM and were statistically analyzed using Kruskal–Wallis test with multiple comparisons (Dunn’s test). *p < 0.05 and ***p < 0.001 as compared to HeLa cells expressing G-GECO1.2-Orai1α and TRPC1

Alternatively, we have analyzed the proximity between TRPC1 and Orai1α by looking for the influence of Ca^2+^ influx through TRPC1 on the fluorescence of G-GECO1.2 fused to Orai1α (G-GECO-Orai1α). G-GECO–Orai1α was transiently co-transfected with STIM1 and TRPC1 or dnTRPC1 mutant into HeLa cells and imaged using an epifluorescence inverted microscope. We have previously reported that G-GECO–Orai1α exhibited diffuse expression in the plasma membrane [30]. Cells were stimulated with 3 µM histamine to elicit Ca^2+^ influx (detected as fluctuations) through Orai1α in the presence of 1 mM extracellular Ca^2+^. Traces from five representative cells are shown in Fig. 3a–d. An average of 87% of HeLa cells expressing STIM1, G-GECO–Orai1α and TRPC1 stimulated with histamine responded with G-GECO1.2 fluorescence fluctuations (Fig. 3e), with an average of 11.7 ± 1.1 fluctuations/10 min (Fig. 3h). Of the cells that did not fluctuate, 11% responded with a sustained plateau and the remaining 2% of cells did not respond (Fig. 3f, g). Substitution of TRPC1 by dnTRPC1 significantly decreased the percentage of fluctuating cells to 58%, while the number of G-GECO1.2 fluorescence fluctuations per cell was unaffected (Fig. 3e–h).

In cells expressing TRPC1, the overall Ca^2+^ influx detected by G-GECO1.2 was significantly greater than that observed in cells expressing dnTRPC mutant (Fig. 3i–k; *p* < 0.05). These findings suggest that either TRPC1 enhances Ca^2+^ influx through Orai1α or that both channels are very close so that Ca^2+^ entry through TRPC1 might be detected by Orai1α-fused G-GECO1.2. Concerning the first hypothesis, it is well established that Orai1/STIM1 forms the primary SOCE channel and leads to the initial Ca^2+^ influx upon depletion of the intracellular Ca^2+^ stores, while TRPC1, can be recruited to ER–PM junctions and inserted into the PM by a mechanism that requires Orai1-mediated Ca^2+^ entry [1]. According to this, it is quite unlikely that TRPC1 modulates the onset of Ca^2+^ influx via Orai1α, but once cation entry through TRPC1 is initiated, there are several mechanisms that might negatively influence Orai1α function, including changes in the driving force for Ca^2+^ entry by membrane depolarization, competition for STIM1 units or the facilitation of Ca^2+^-dependent Orai1α inactivation [34]. Accordingly, we have investigated whether TRPC1 influences the phosphorylation of Orai1α at serine residues, which has been demonstrated to induce Orai1 channel inactivation [4, 35]. To investigate this phenomenon, HeLa cells were co-transfected with STIM1, Orai1α and TRPC1 or the dnTRPC1 mutant. Cells were subsequently stimulated with histamine in the presence of 1 mM extracellular Ca^2+^ and Orai1α serine phosphorylation was estimated 10 s, 1 min and 10 min after the addition of the agonist. As depicted in Fig. S6a, Western blot analysis reveals that Orai1α serine phosphorylation was comparable in cells expressing TRPC1 and dnTRPC1 mutant, which indicates that Ca^2+^ influx via TRPC1 does not alter serine phosphorylation-dependent inactivation of Orai1α. We have further investigated whether TRPC1 might influence the plasma membrane expression of Orai1α by biotinylation. HeLa cells were co-transfected with STIM1, Orai1α and either TRPC1, dnTRPC1 or shTRPC1. Cells were stimulated with histamine (3 µM) in the presence of 1 mM extracellular Ca^2+^ and the exposure of Orai1α in the plasma membrane was estimated 10 s, 1 min and 10 min after the addition of the agonist. As depicted in Fig. S6b, our results indicate that the plasma membrane location of Orai1α was similar in cells expressing TRPC1 or the dnTRPC1, as well as in cells transfected with shTRPC1, thus suggesting that TRPC1-mediated cation entry does not influence the plasma membrane expression of Orai1α. Altogether, these findings challenge a positive role for TRPC1 in Ca^2+^ influx via Orai1α and further support that Orai1α and TRPC1 are in the close proximity.

To further explore whether Ca^2+^ entry via TRPC1 can influence G-GECO1.2 fluorescence, we repeated the experiments in cells expressing G-GECO fused to the dominant-negative Orai1α-E106Q mutant (GECO-dnOrai1α), which lacks Ca^2+^ influx through the channel. It is noteworthy that under these conditions, TRPC1 is not expected to be fully functional as Ca^2+^ entry through Orai1α is impaired [1]. Even with this limitation, when cells were co-transfected with STIM1, TRPC1 and GECO–dnOrai1α, the percentage of cells with fluctuations in G-GECO1.2 fluorescence and the magnitude of Ca^2+^ influx were significantly greater than in cells expressing dnTRPC1 (*p* < 0.05; Fig. 3e–k). These findings indicate that Ca^2+^ entry via TRPC1 influences the fluorescence of G-GECO1.2 associated to Orai1α, which confirms that Orai1α and TRPC1 are adjacent.

### Orai1α modulates cation entry through TRPC1

**Fig. 4:**
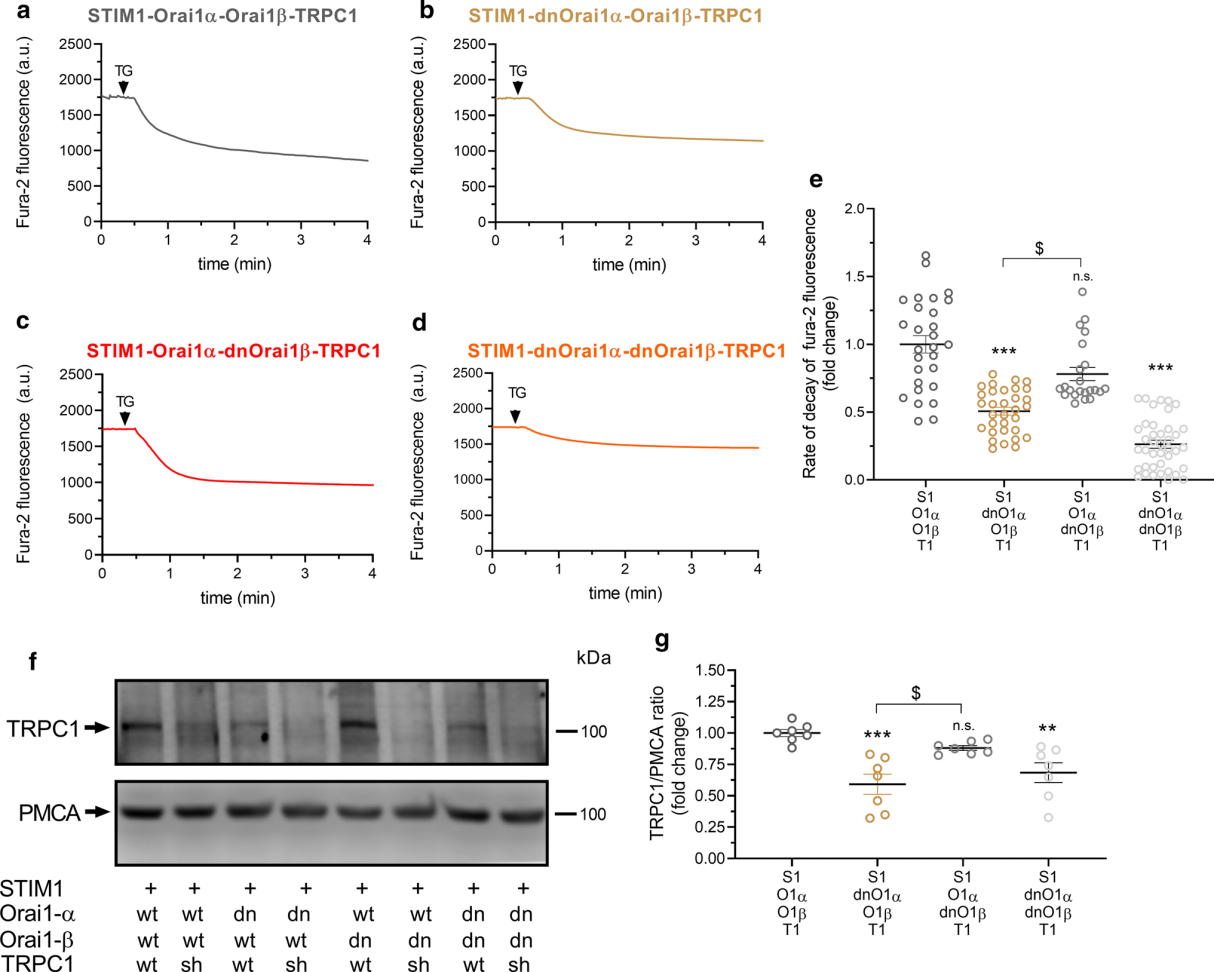
Orai1α modulates Mn^2+^ influx through TRPC1 and TRPC1 plasma membrane expression. **a**–**d** Representative responses to 2 µM TG in HeLa cells co-transfected with STIM1, Orai1α, Orai1β and TRPC1 or the corresponding dominant-negative Orai1 mutants, as described. Cells were super-fused with HBSS containing 0.5 mM Mn^2+^ and 1 mM Ca^2+^ and stimulated with 2 µM TG (indicated by arrow). Fura-2 fluorescence was measured at an excitation wavelength of 360 nm, the isoemissive wavelength. Representative traces were chosen to represent the datasets. **e** Quantification of the rate of decay of fura-2 fluorescence under the different experimental conditions (from left to right, *n* = 28, 32, 22 and 40; *n* values correspond to individual cells). Scatter plots are represented as mean ± SEM and were statistically analyzed using Kruskal–Wallis test with multiple comparisons (Dunn’s test). **p < 0.01 and ***p < 0.001 as compared to HeLa cells expressing STIM1, Orai1α, Orai1β and TRPC1. ^$^*p* < 0.05, statistical significance among the responses observed in cells expressing dnOrai1α or dnOrai1β mutants. **f** HeLa cells were co-transfected with STIM1-CFP, Orai1α (or dnOrai1α mutant, as indicated), Orai1β (or dnOrai1β mutant, as indicated) and TRPC1 (or with shTRPC1, as indicated). Forty-eight hours later, cells were mixed with biotinylation buffer containing EZ-Link sulfo-NHS-LC-biotin, and cell surface proteins were labeled by biotinylation, as described in “Material and methods”. Labeled proteins were pulled down with streptavidin-coated agarose beads. The pellet (containing the plasma membrane fraction) was analyzed by SDS-PAGE and Western blotting using anti-TRPC1 or anti-PMCA antibody, as indicated. Molecular masses indicated on the right were determined using molecular-mass markers run in the same gel. These results are representative of seven separate experiments. **g** Quantification of TRPC1 plasma membrane expression under the different experimental conditions normalized to the PMCA expression. Scatter plots are represented as mean ± SEM and were statistically analyzed using Kruskal–Wallis test with multiple comparisons (Dunn’s test). **p < 0.01 and ***p < 0.001 as compared to HeLa cells expressing STIM1, Orai1α, Orai1β and TRPC1. ^$^*p* < 0.05, statistical significance among the TRPC1 surface expression in cells expressing dnOrai1α or dnOrai1β mutants

To investigate the possible role of Orai1 variants in the modulation of TRPC1 function, HeLa cells were co-transfected with STIM1, TRPC1, Orai1α and Orai1β, or their respective dominant-negative mutants: dnOrai1α and dnOrai1β. Mn^2+^ was used to evaluate the effect of Orai1α and Orai1β on TG-evoked bivalent-cation influx through TRPC1 as Orai1 has been reported to be poorly permeable to Mn^2+^ [36] and highly selective for Ca^2+^ in the presence of divalent cations [37]. This cation can be used as a surrogate for Ca^2+^ entry, given its quenching effect on fura-2 [38]. Cells were super-fused with a medium containing 0.5 mM Mn^2+^ and 1 mM Ca^2+^ and stimulated with 2 µM TG. Fura-2 was excited at the isoemissive wavelength, 360 nm, to monitor the quenching of fluorescence by Mn^2+^. As shown in Fig. 4a, in cells co-transfected with STIM1, Orai1α, Orai1β and TRPC1, addition of TG resulted in a sustained quenching of fura-2 fluorescence. Transient expression of STIM1, TRPC1, Orai1α and Orai1β significantly enhanced TG-evoked quenching of fura-2 fluorescence as compared to mock-treated cells (Fig. S7). As shown in Fig. S8, co-expression of STIM1 and Orai1α or Orai1β did not significantly enhance Mn^2+^ entry upon treatment with TG as compared to mock-treated cells, which indicates that Mn^2+^ enters the cell through TRPC1. Co-transfection of dnOrai1α, instead of WT Orai1α, resulted in an attenuation in the rate of Mn^2+^ entry (Fig. 4b). The initial rate of Mn^2+^-evoked fluorescence quenching in cells expressing dnOrai1α was significantly decreased to approximately 50% of control (Fig. 4e; *p* < 0.001). By contrast, cells co-expressing STIM1, TRPC1, Orai1α and dnOrai1β exhibited a similar response to TG than those co-expressing Orai1β (Fig. 4c vs a), which strongly suggests that functional Orai1β is not required for TRPC1-mediated cation entry. Further substitution of both, Orai1α and Orai1β, for their respective pore-dead mutants significantly attenuated the rate of Mn^2+^ entry and the overall quenching of fura-2 fluorescence (Fig. 4d, e; *p* < 0.001), although the effect of co-expression of both dominant-negative mutants was not found to be significantly different from that induced by dnOrai1α (Fig. 4e). These findings indicate that Orai1α, but not Orai1β, is required for TRPC1 function.

We have noticed that our findings in HeLa cells differ from those reported by Desai et al. in HEK-293 cells, which showed that both Orai1α- and Orai1β-mediated signals could activate TRPC1 [15]. Therefore, we have repeated the experimental procedure used in HeLa cells to assess the role of Orai1α and Orai1β on TRPC1 activation in HEK-293 cells and we have found that, in these cells, co-expression of STIM1, Orai1 (EYFP-Orai1 plasmid, which might give Orai1α and Orai1β), and TRPC1 significantly enhances TG-evoked Mn^2+^ entry (Fig. S9a, b). This response was abolished when Orai1 was substituted by a dominant-negative Orai1 mutant (Fig. S9c). As depicted in Fig. S9d–f, and in agreement with Desai et al. [15], both Orai1α and Orai1β enhanced TG-evoked Mn^2+^ equally well, and generated signals comparable to those observed with wild-type Orai1, which confirms that, in HEK-293 cells, Orai1α and Orai1β are equally effective in activating TRPC1. Interestingly, these findings indicate intercellular differences in the functional role of Orai1α and Orai1β.

Next, we investigated the mechanism underlying the modulation of TRPC1 by Orai1α in HeLa cells. As TRPC1 has been recruited to the PM by a mechanism dependent on Orai1 [1], we have explored whether the Orai1 variants play a relevant role in the surface exposure of TRPC1. HeLa cells were co-transfected with STIM1, TRPC1, Orai1α and Orai1β or their respective dominant-negative mutants. In some experiments, cells were co-transfected with shTRPC1 instead of TRPC1 expression plasmid to assess specificity of the antibody. As shown in Fig. 4f, TRPC1 was clearly detected by biotinylation in the PM of cells expressing STIM1, TRPC1, Orai1α and Orai1β. Substitution of Orai1α for dnOrai1α significantly attenuated the amount of TRPC1 detected in the plasma membrane (Fig. 4f, g; *p* < 0.001). Consistent with the results presented in Fig. 4e, co-expression of dnOrai1β instead of Orai1β did not significantly attenuate the expression of TRPC1 in the PM and substitution of both Orai1 variants for their respective dominant-negative mutants significantly attenuated TRPC1 exposure in the PM and led to results comparable to that observed in cells expressing dnOrai1α (Fig. 4f, g; *p* < 0.01). In cells transfected with shTRPC1, the expression of TRPC1 in the PM was undetectable (Fig. 4f).

**Fig. 5:**
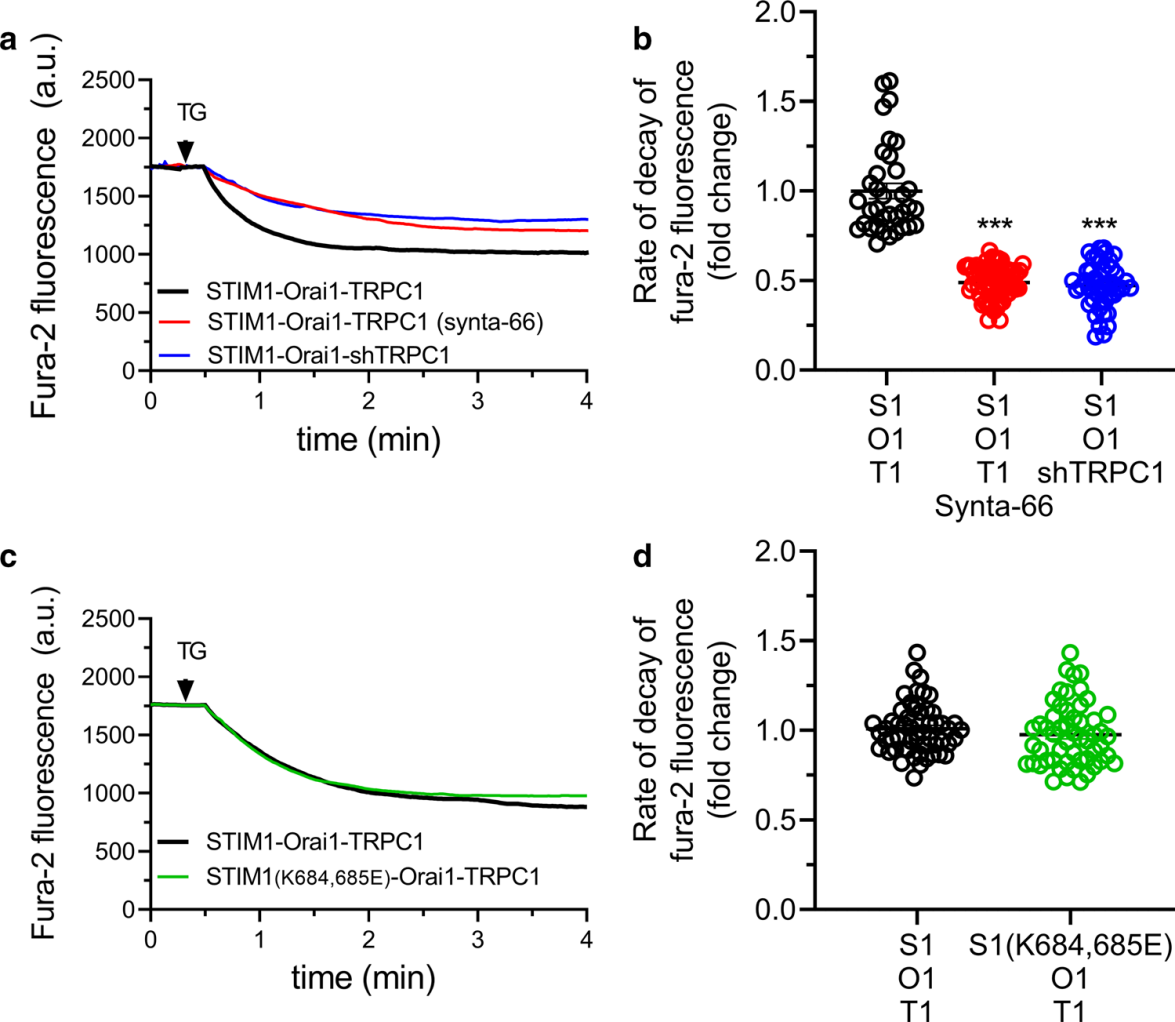
Effect of Orai1 inhibition and expression of the STIM1 (K684,685E) mutant on Mn^2+^ influx in HeLa cells. **a** Representative responses to 2 µM TG in HeLa cells co-transfected with STIM1, Orai1 and TRPC1, in the absence or presence of synta66 (10 µM), or co-transfected with STIM1, Orai1 and shTRPC1, as described. Cells were super-fused with HBSS containing 0.5 mM Mn^2+^ and 1 mM Ca^2+^ and stimulated with 2 µM TG (indicated by arrow). Fura-2 fluorescence was measured at an excitation wavelength of 360 nm, the isoemissive wavelength. Representative traces were chosen to represent the datasets. **b** Quantification of the rate of decay of fura-2 fluorescence under the different experimental conditions (from left to right, *n* = 35, 61 and 47; *n* values correspond to individual cells). Scatter plots are represented as mean ± SEM and were statistically analyzed using Kruskal–Wallis test with multiple comparisons (Dunn’s test). ***p < 0.001 as compared to HeLa cells expressing STIM1, Orai1 and TRPC1. **c** Representative responses to TG in HeLa cells co-transfected with STIM1 or the STIM1(K684,685E) mutant, Orai1 and TRPC1, as indicated. Cells were super-fused with HBSS containing 0.5 mM Mn^2+^ and 1 mM Ca^2+^ and stimulated with 2 µM TG (indicated by arrow). Fura-2 fluorescence was measured at an excitation wavelength of 360 nm, the isoemissive wavelength. Representative traces were chosen to represent the datasets. **d** Quantification of the rate of decay of fura-2 fluorescence under the different experimental conditions (from left to right, *n* = 55 and 54; *n* values correspond to individual cells). Scatter plots are represented as mean ± SEM and were statistically analyzed using Mann–Whitney *U* test

We have further evaluated the role of Orai1 in TG-induced Mn^2+^ entry by TRPC1 in HeLa cells using the Orai1-specific inhibitor synta66. As shown in Fig. 5a, b, Mn^2+^ entry was significantly inhibited in cells treated with 10 µM synta66 to a similar extent that in cells where TRPC1 has been knocked down, which further indicates that Mn^2+^ entry by TRPC1 requires functional Orai1. We have further analyzed whether TRPC1 activation by Orai1α further depends on STIM1. To test this issue, HeLa cells were co-transfected with STIM1, Orai1 and TRPC1 or with the STIM1(K684,685E) mutant (which is unable to activate TRPC1 [39, 40]), Orai1 and TRPC1. As depicted in Fig. 5c, d, Mn^2+^ entry evoked by TG was unaltered in cells expressing the STIM1(K684,685E) mutant, thus suggesting that STIM1 is required for the activation of Orai1, but then, it does not seem to be further required to gate TRPC1 channels.

## Discussion

Our present studies reveal that Orai1α co-localizes with TRPC1 and is required for the location of this channel in the plasma membrane and its activation upon Ca^2+^ store depletion or cell stimulation with physiological agonists. First, we have found interaction between Orai1α and TRPC1 in native cells by co-immunoprecipitation using both anti-Orai1 and anti-TRPC1 antibodies. Second, as antibody-based approaches strongly depend on the specificity of the antibodies themselves, we have further demonstrated co-localization of Orai1α and TRPC1 by an independent mechanism using APEX2 proximity labeling assay in cells expressing STIM1-CFP, TRPC1 and Orai1α-GFP. These approaches indicate co-localization of Orai1α and TRPC1 under resting conditions and that this interaction significantly enhances upon Ca^2+^ store depletion using TG or histamine. Nevertheless, our findings suggest that Orai1β is scarcely associated to TRPC1 under resting conditions but this interaction, if significant, is constitutive and does not depend on Ca^2+^ store depletion or stimulation with agonists.

Our results provide further evidence for the co-localization between Orai1α and TRPC1 by revealing cross-detection of TRPC1-mediated Ca^2+^ entry by G-GECO–Orai1α. GECO is a channel-fused fluorescent Ca^2+^ indicator that detects changes in Ca^2+^ concentration in the close proximity of the channel to which it is associated [41]. We have previously found that G-GECO–Orai1α is unable to detect changes in the Ca^2+^ concentration near the channel upon discharge of the endoplasmic reticulum using TG in HeLa cells [30], thus suggesting that TRPC1 should be close enough to Orai1α to influence the signal of the fluorophore. Altogether, using three different experimental approaches, our results provide strong evidence for the co-localization of Orai1α and TRPC1, an event that is regulated by Ca^2+^ store depletion or stimulation with physiological agonists.

An additional important finding of our study is that TRPC1 plasma membrane location and function is strongly dependent on the functional expression of Orai1α, as expression of the dominant-negative Orai1α mutant (Orai1αE106Q-EGFP), instead of functional Orai1α, significantly impaired both the surface exposure of TRPC1 and divalent cation entry through the channel. In support of these findings, inhibition of Orai1 by synta66 significantly attenuated Mn^2+^ entry to a similar extent to TRPC1 knockdown in HeLa cells. The experiments were performed in the presence of 1 mM extracellular Ca^2+^ to maintain Orai1 Ca^2+^ selectivity [8] and allow Orai1-mediated Ca^2+^ entry-dependent responses, such as TRPC1 expression in the plasma membrane [1]. Consistent with this observation, in HeLa cells co-expressing dnOrai1α, the magnitude of histamine-evoked Ca^2+^ responses was similar either expressing TRPC1 or the dominant-negative TRPC1 mutant (see Fig. 1), which further confirms that functional Orai1α is essential for TRPC1 channel function. By contrast, our results indicate that the short Orai1 variant, Orai1β, has a negligible effect, if any, on the plasma membrane location and function of TRPC1. These findings are consistent with previous studies reporting that location of TRPC1 in the plasma membrane depends on Ca^2+^ influx through Orai1 [26] and further identifies the Orai1 variant involved in this process. Furthermore, this observation reveals important functional differences between both Orai1 variants. The role of Orai1α, but not Orai1β, in TRPC1 plasma membrane expression in HeLa cells might be associated to the presence exclusively in Orai1α of a caveolin-binding domain, which allows Orai1α interact to caveolin [42]. Caveolin-1 has also been reported to mediate trafficking of TRPC1 to the plasma membrane, which may be required for SOCE [43]. As TRPC1 trafficking to the plasma membrane requires Ca^2+^ entry via Orai1 [44] and both Orai1α and TRPC1 co-localize with caveolin-1, it is expected that Orai1α plays a predominant role triggering TRPC1 trafficking to the plasma membrane in HeLa cells. In addition to the caveolin-binding domain, other N-terminal functional motifs of Orai1α, missing in Orai1β, might lead to the recruitment of TRPC1 in the plasma membrane by Orai1α, presumably in the lipid rafts, where TRPC1 has been located [45]. For instance, the AC8-binding domain, which is responsible for the interaction of Orai1α with AC8 in lipid raft domains [46, 47] might play a relevant role in the location and recruitment of signaling proteins associated to the Orai1α channelosome, including TRPC1.

Upon stimulation with physiological concentrations of agonists, cells develop repetitive oscillations in the concentration of cytosolic Ca^2+^ whose frequency and intensity have been reported to play a key role in the development of a number of cellular events, such as gene transcription [48]. Ca^2+^ oscillations are triggered by IP_3_-evoked Ca^2+^ release from the intracellular Ca^2+^ stores leading to the activation of store-operated channels in the plasma membrane [5, 11]. Using a four-fold co-expression of STIM1, Orai1α, Orai1β and TRPC1 or a triple co-expression combination of STIM1, Orai1α or Orai1β and TRPC1, we provide evidence supporting that both, Orai1α and Orai1β, are required to sustain Ca^2+^ oscillations, while TRPC1 has a minor role, if any. While TRPC1 does not seem to play a relevant role in Ca^2+^ oscillations, recruitment of this channel clearly enhances the magnitude of Ca^2+^ responses (observed in cells expressing functional Orai1α (see Figs. 1m and S4m), which further confirms that TRPC1 function depends on Ca^2+^ influx via Orai1α). The observation that TRPC1 is not required for Ca^2+^ oscillations cannot be attributed to a loss of function of transiently expressed TRPC1 due to ectopic location or any other artifact as co-expression of STIM1, Orai1α, Orai1β and TRPC1 in HeLa cells resulted in a robust Mn^2+^ entry upon stimulation with TG in the presence of extracellular Ca^2+^.

In conclusion, our results provide multiple evidence for store depletion-dependent co-localization of Orai1α with TRPC1 and the regulation of TRPC1 plasma membrane expression and channel function by Orai1α; by contrast, the short Orai1 variant, Orai1β, is not required for plasma membrane expression and function of TRPC1. The approaches used do not allow sufficient resolution to discriminate if Orai1α and TRPC1 interact directly or they are located in the same cellular nano-domain as near components of a functional protein complex. Our results provide evidence that Orai1α and Orai1β are non-redundant and might display differential functional roles in calcium signaling.

### Supplementary Information

Below is the link to the electronic supplementary material.

Supplementary file1 (DOCX 2895 KB)

## Data Availability

All data are available in the main text or the supplementary materials.
