# Supplementary material for: Orai1α, but not Orai1β, co-localizes with TRPC1 and is required for its plasma membrane location and activation in HeLa cells
Source: Cell Mol Life Sci. 2022 Jan 6;79(1):33. doi: 10.1007/s00018-021-04098-w (PMC8732813; doi:10.1007/s00018-021-04098-w)
Supplement: Supplementary file 1 — Supplementary file1 (DOCX 2895 KB) [file 18_2021_4098_MOESM1_ESM.docx]

Supplementary Materials

**Orai1α, but not Orai1β, co-localizes with TRPC1 and is required for its plasma membrane location and activation**

Jose Sanchez-Collado,**^1^**^†^ Jose J. Lopez,**^1^**^†^***** Isaac Jardin,**^1^** Alejandro Berna-Erro,**^1^** Pedro J. Camello,**^2^** Carlos Cantonero,**^1^** Tarik Smani,**^3,4^** Gines M. Salido,**^1^** Juan A. Rosado**^1^***

*Corresponding author. Email: [jarosado@unex.es,](mailto:jarosado@unex.es,) jjlopez@unex.es

**This PDF file includes:**

Figs. S1 to S9

**
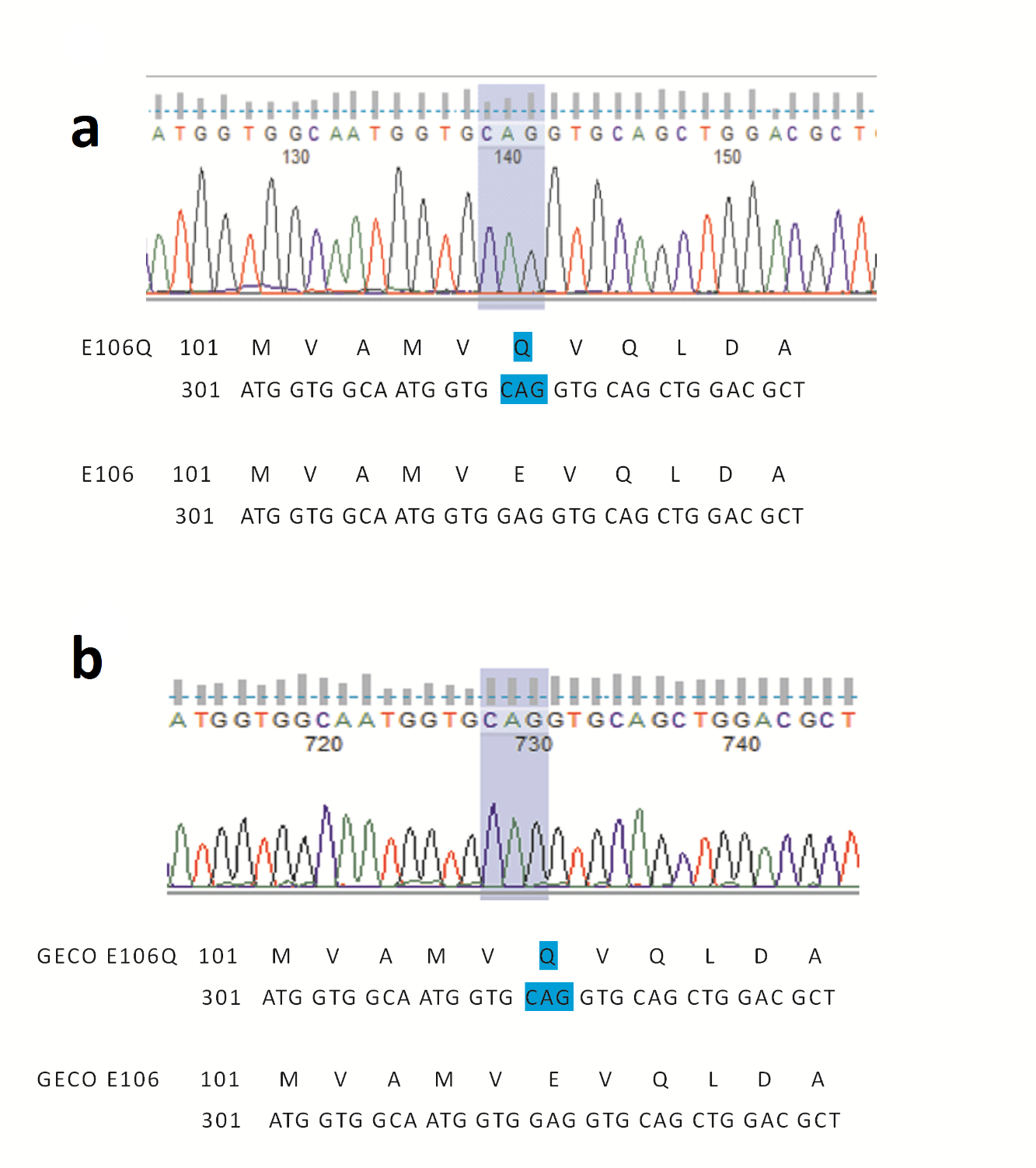
**

**Figure S1.** Sequencing results of Orai1βE43Q-EGFP (corresponding to the E106Q mutant of the Orai1α variant) **(a)** and GECO-Orai1E106Q mutants **(b)**.

**
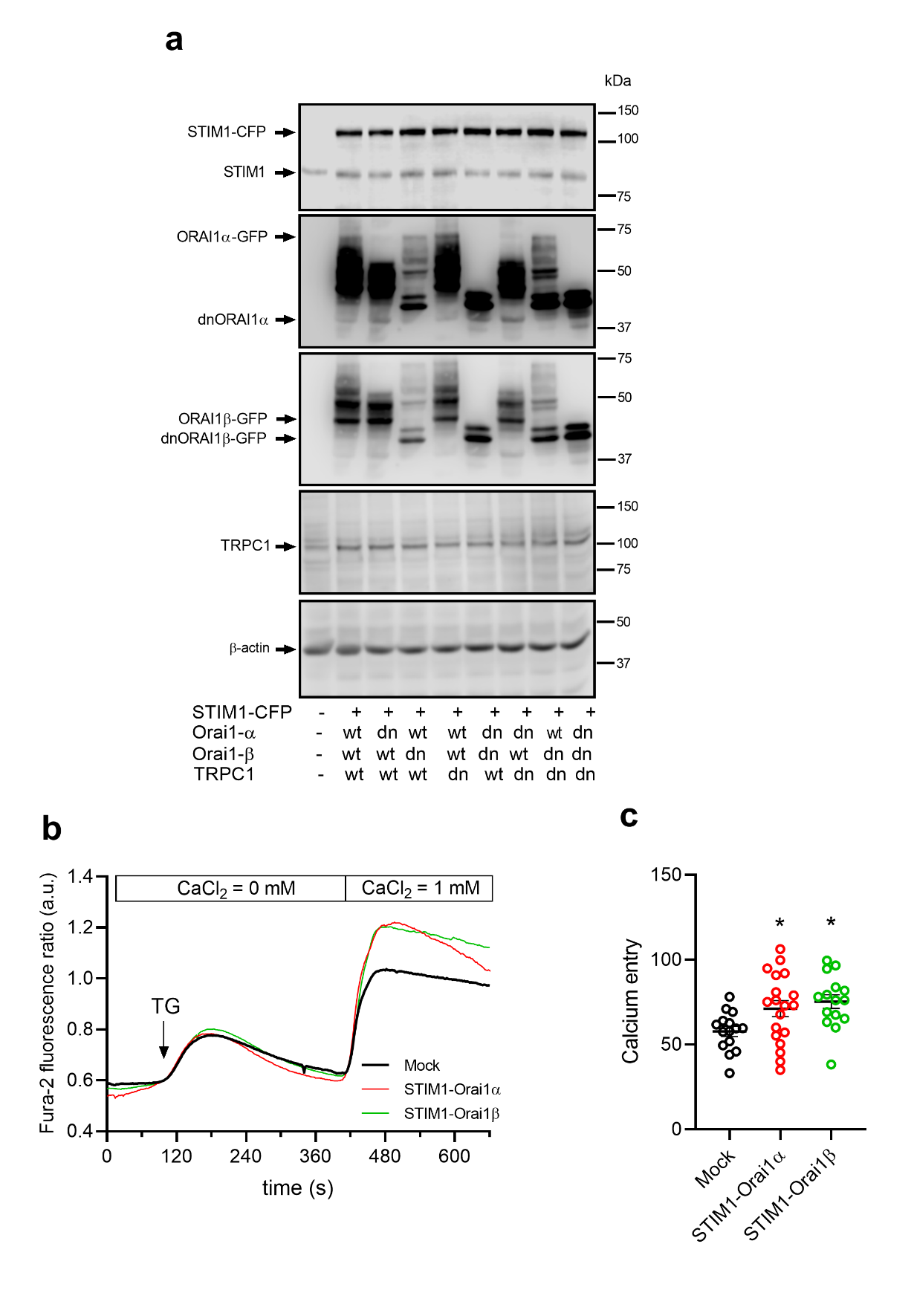
**

**Figure S2. STIM1, Orai1 variants/mutants and TRPC1 expression in HeLa cells.**

**a** HeLa cells were co-transfected with STIM1-CFP, Orai1α-GFP (or dnOrai1α mutant, as indicated), Orai1β-GFP (or dnOrai1β-GFP mutant, as indicated) and TRPC1. Forty-eight hours later cells were lysed and subjected to 10% SDS-PAGE and Western blotting with anti-STIM1 antibody, anti-Orai1 C-terminal antibody or anti-TRPC1 antibody, as described in Material and Methods. Membranes were reprobed with anti-β-actin antibody for protein loading control. Molecular masses indicated on the right were determined using molecular-mass markers run in the same gel. Blots are representative of three separate experiments. **b** HeLa cells were co-transfected with STIM1 and Orai1α, STIM1 and Orai1β or empty vector (mock). Fura-2-loaded cells were perfused with a Ca^2+^-free medium (250 μM EGTA added) and then stimulated with TG (1 μM) followed by reintroduction of external Ca^2+^ (final concentration 1 mM) to initiate Ca^2+^ entry. **c** Quantification of Ca^2+^ entry estimated as described in Material and Methods. Scatter plots are represented as mean ± SEM and were statistically analyzed using Kruskal–Wallis test with multiple comparisons (Dunn´s test). **p* < 0.05 as compared to mock-treated cells.


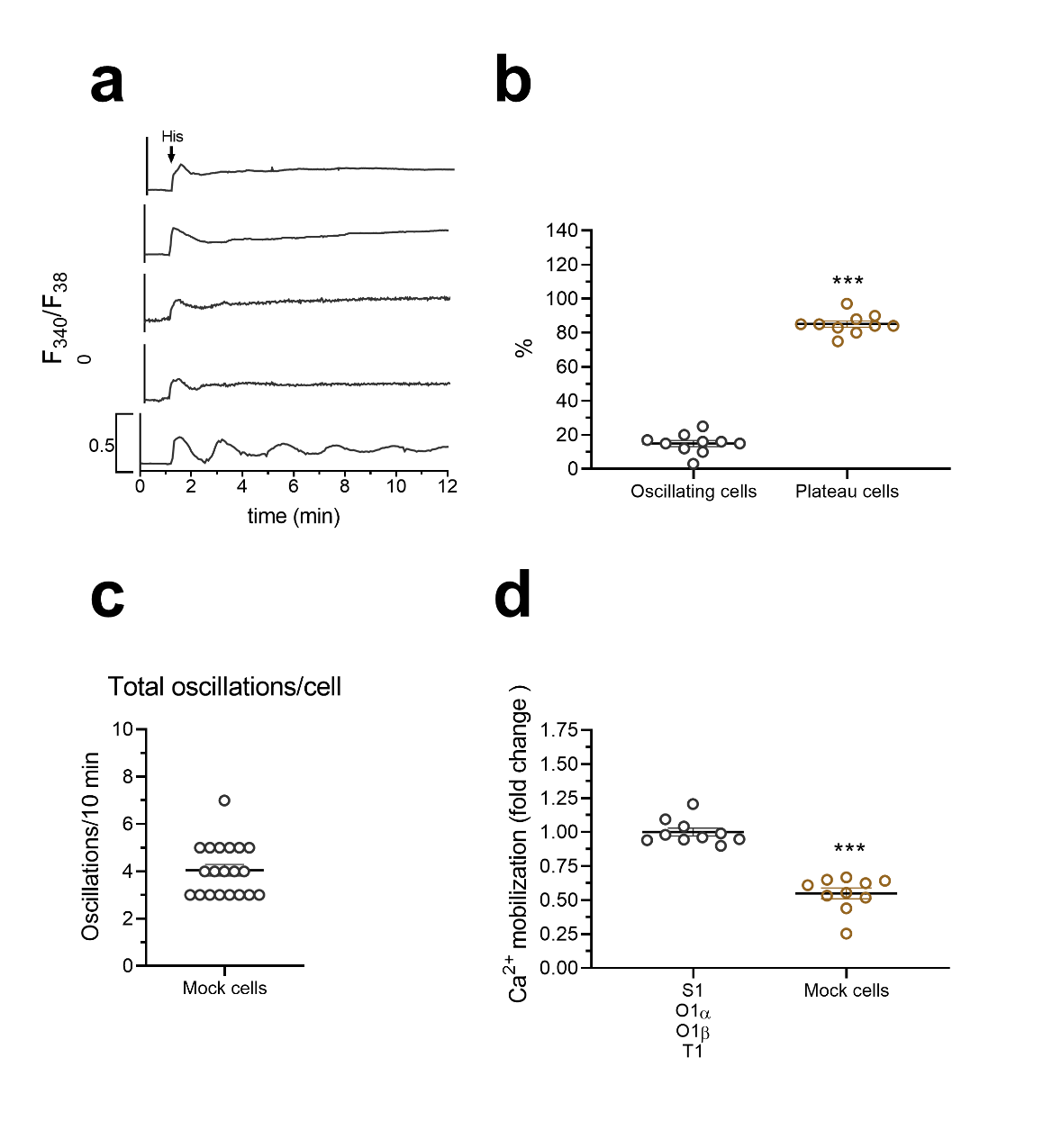


**Figure S3.** **Histamine-induced Ca^2+^ oscillations in mock-treated HeLa cells.**

**a** Representative Ca^2+^ oscillations in response to 3 µM histamine measured using
fura-2 in HeLa cells not incubated with plasmids but otherwise treated as cells in Figure 1. Cells were superfused with HBSS containing 1 mM Ca^2+^ and stimulated with 3 µM histamine at 1 min (indicated by arrow). Representative traces from five cells were chosen to represent the datasets. **b-c** Quantification of the percentage of oscillating and plateau cells  **(b)** and total oscillations/cell in 10 min **(c)** for data presented in **a** (for **b**, n = 10; n-values correspond to independent experiments; for **C** n=24; n-values correspond to individual cells). **d** Quantification of Ca^2+^ mobilization estimated in mock-treated cells in comparison to cells expressing STIM1, Orai1α, Orai1β and TRPC1 (data from Fig. 1). Scatter plots are represented as mean ± SEM and were statistically analyzed using Mann–Whitney U test to HeLa cells expressing STIM1, Orai1α, Orai1β and TRPC1 (****p* < 0.001).

**
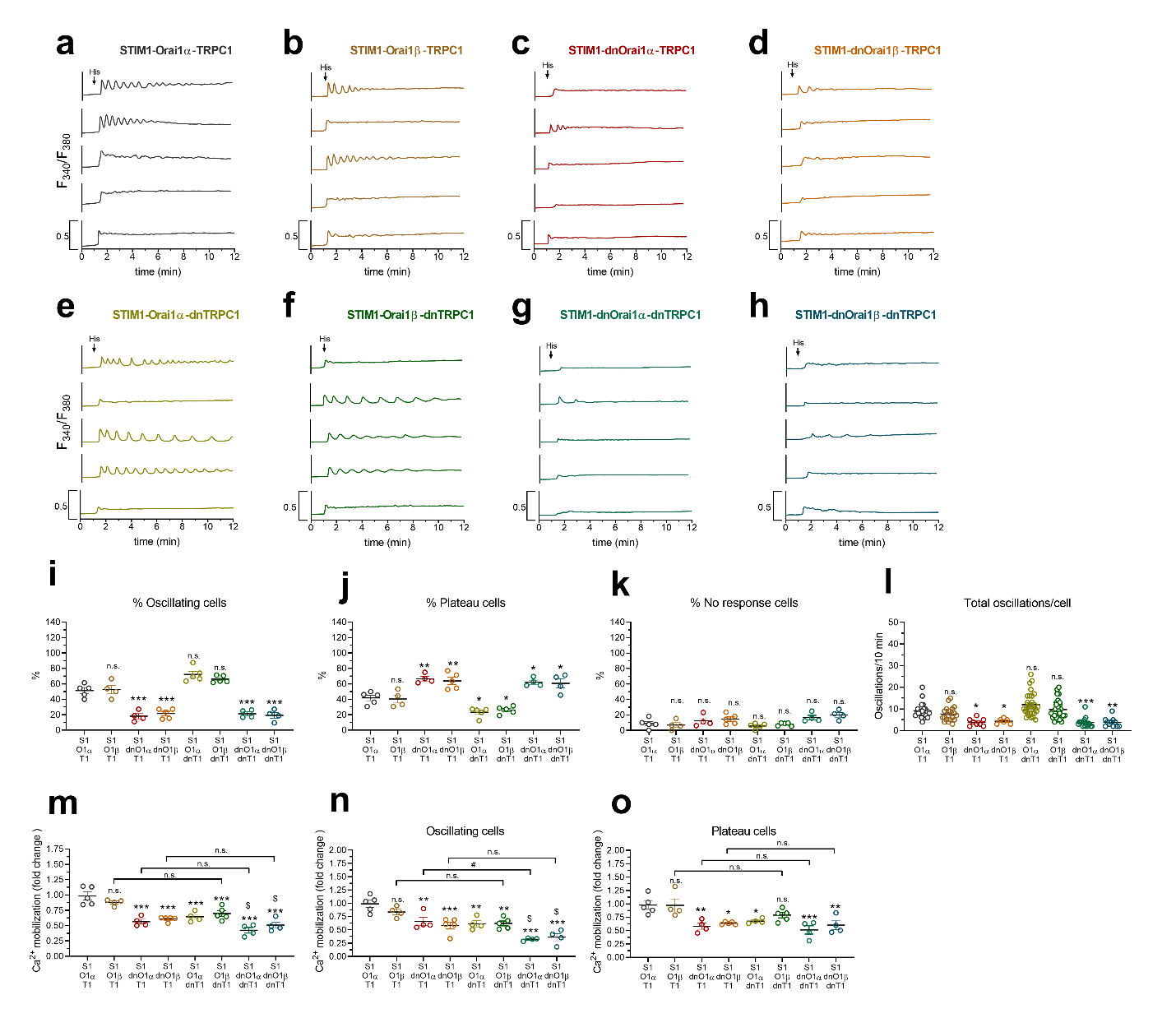
**

**Figure S4.** **Orai1α and Orai1β, but not TRPC1, are required for histamine-induced Ca^2+^ oscillations.**

**a-h** Representative Ca^2+^ oscillations in response to 3 µM histamine measured using
fura-2 in HeLa cells co-transfected with STIM1, Orai1α or Orai1β and TRPC1 or the corresponding dominant negative mutants, as described. Cells were superfused with HBSS containing 1 mM Ca^2+^ and stimulated with 3 µM histamine at 1 min (indicated by arrow). Representative traces from five cells/condition were chosen to represent the datasets. **i-l** Quantification of the percentage of oscillating cells **(i)**, percentage of plateau cells **(j)**, percentage of non-responding cells **(k)** and total oscillations/cell in 10 min **(l)** for data presented in **a-h**(for **i** to **k**, n = 4-5; n-values correspond to independent experiments; for **l**, from left to right, n=22, 20, 8, 6, 30, 28, 16 and 11; n-values correspond to individual cells). **m-o** Quantification of Ca^2+^ mobilization for all the conditions from **a** to **h** estimated in all the cells **(m)**, oscillating cells **(n)** and plateau cells **(o)**. Scatter plots are represented as mean ± SEM and were statistically analyzed using Kruskal–Wallis test with multiple comparisons (Dunn´s test) to HeLa cells expressing STIM1, Orai1α or Orai1β and TRPC1 (**p* < 0.05 and ****p* < 0.001), HeLa cells expressing STIM1, Orai1α or Orai1β and dnTRPC1 (for conditions including the expression of dnTRPC1; ^$^*p* < 0.05 and ^$$^*p* < 0.01) or the corresponding condition with WT TRPC1 vs dnTRPC1 (^#^*p* < 0.05).


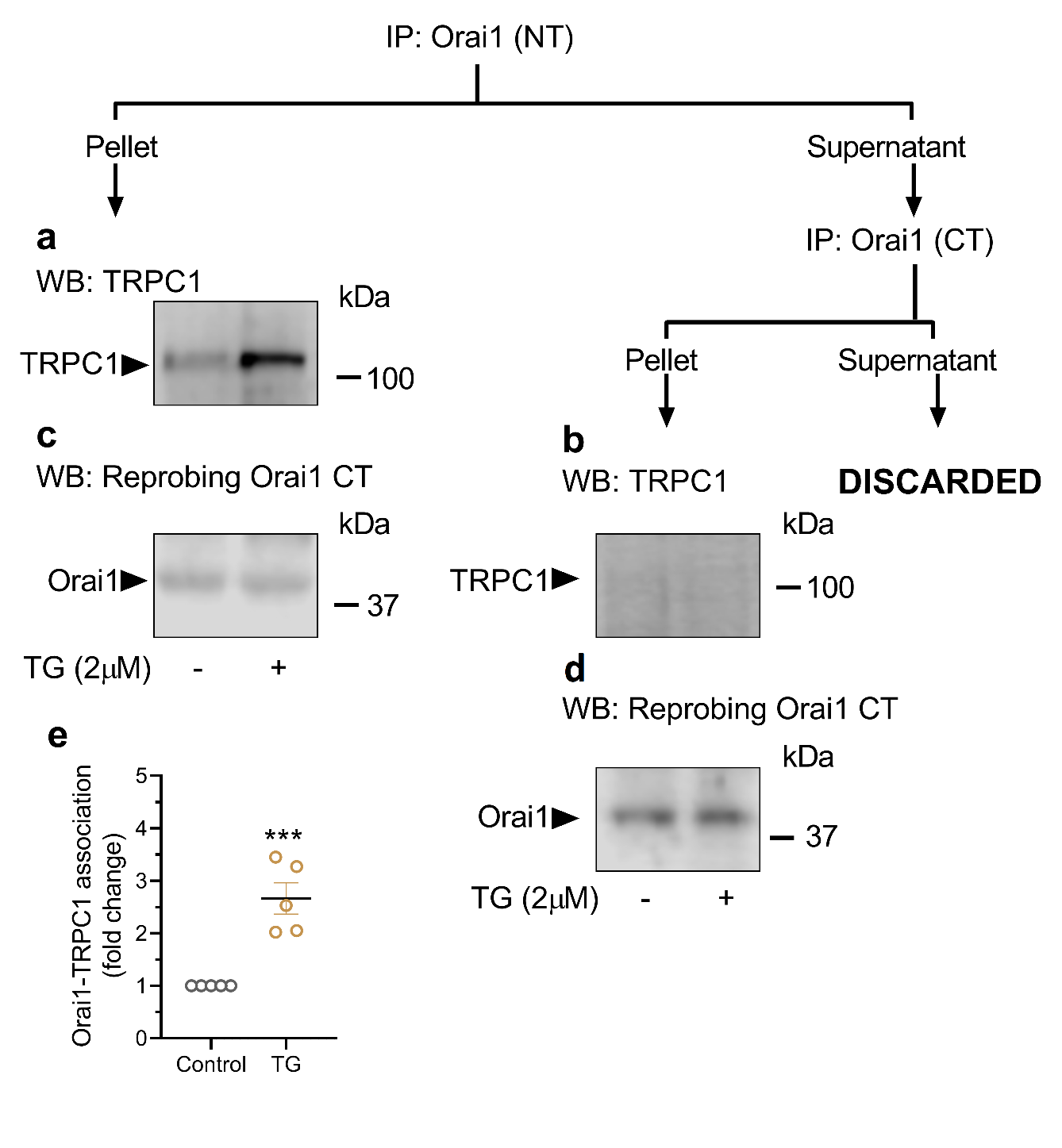


**Figure S5. TRPC1 interacts exclusively with Orai1α.**

HeLa cells were suspended in HBS containing 1 mM Ca^2+^ and then stimulated for 1 min with 2 µM TG or the vehicle and lysed. Whole-cell lysates were immunoprecipitated with anti-Orai1 antibody (epitope N-terminal (NT): amino acids 2-61). The immunoprecipitates (pellet) were then subjected to 10% SDS-PAGE and Western blotting with the anti-TRPC1 antibody **(a)**, as described in Material and Methods. Membranes were reprobed with the anti-Orai1 antibody (epitope C-terminal (CT): amino acids 288-301) for protein loading control **(c)**. The supernatant of the immunoprecipitation with anti-Orai1 NT-antibody was further immunoprecipitated with the anti-Orai1 CT-antibody. The pellet was subjected to 10% SDS-PAGE and Western blotting with the anti-TRPC1 antibody **(b)** and membranes were reprobed with the anti-Orai1 CT-antibody for protein loading control **(d)**. Molecular masses indicated on the right were determined using molecular-mass markers run in the same gel. Blots are representative of five separate experiments. **e** Quantification of TRPC1-Orai1 association under the different experimental conditions normalized to the Orai1 expression. Scatter plots are represented as mean ± SEM and were statistically analyzed using Mann–Whitney U test. ****p*< 0.001 as compared to Control.


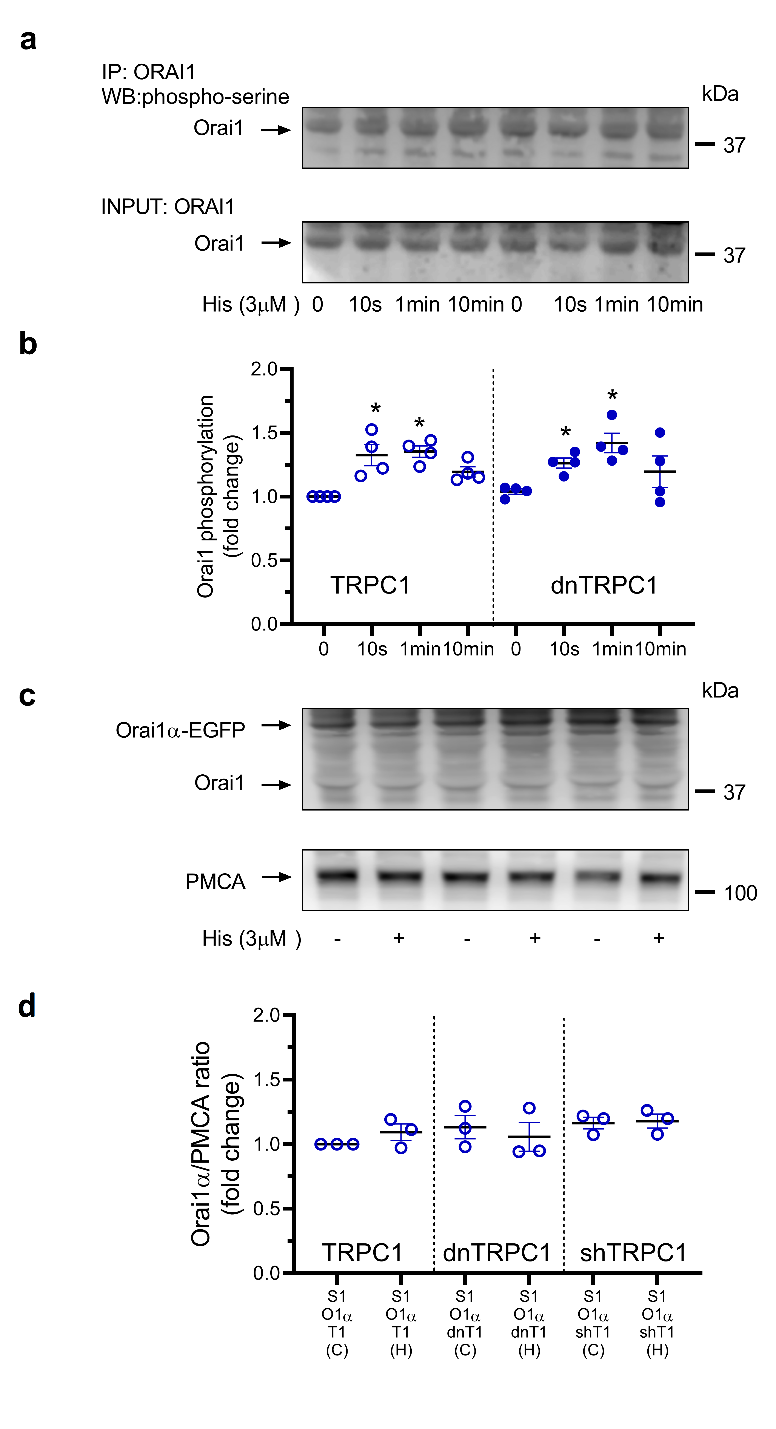


**Figure S6. TRPC1 does not alter either the plasma membrane location or serine phosphorylation of Orai1α.**

**a-b** HeLa cells were co-transfected with STIM1-CFP, Orai1α-GFP and TRPC1 (or dnTRPC1 mutant, as indicated). Forty-eight hours later cells were suspended in HBS containing 1 mM Ca^2+^ and then stimulated with 3 µM histamine. Samples were taken 1s before and 10 s, 1 min and 10 min after the addition of histamine and lysed. Whole-cell lysates were immunoprecipitated with anti-Orai1 C-terminal antibody. The immunoprecipitates were then subjected to 8% SDS-PAGE and Western blotting with specific anti-phosphoserine antibody (**a**, top panel), as described in Material and Methods. Membranes were reprobed with the anti-Orai1 C-terminal antibody for protein loading control (**a**, bottom panel). Molecular masses indicated on the right were determined using molecular-mass markers run in the same gel. **b** Quantification of Orai1α serine phosphorylation under the different experimental conditions normalized to the Orai1α expression. Scatter plots are represented as mean ± SEM, expressed as fold change (experimental/control) and were statistically analyzed using Kruskal–Wallis test with multiple comparisons (Dunn´s test). **p* < 0.05 as compared to Control. **c-d** HeLa cells were co-transfected with STIM1-CFP, Orai1α-GFP and either TRPC1, dnTRPC1 mutant or shTRPC1, as indicated. Forty-eight hours later cells were suspended in HBS containing 1 mM Ca^2+^, stimulated for 1 min with 3 µM histamine or left untreated and mixed with biotinylation buffer containing EZ-Link sulfo-NHS-LC-biotin. Cell surface proteins were labeled by biotinylation as described in Material and Methods. Labeled proteins were pulled down with streptavidin-coated agarose beads. The pellet (containing the plasma membrane fraction) was analyzed by SDS-PAGE and Western blotting using anti-Orai1α (C terminal) or anti-PMCA antibody, as indicated. Molecular masses indicated on the right were determined using molecular-mass markers run in the same gel. These results are representative of 3 separate experiments. **d** Quantification of Orai1α plasma membrane expression under the different experimental conditions normalized to the PMCA expression. Scatter plots are represented as mean ± SEM and expressed as fold change (experimental/control (resting cells co-transfected with STIM1-CFP, Orai1α-GFP and TRPC1)). Data were statistically analyzed using Kruskal–Wallis test with multiple comparisons (Dunn´s test).

**
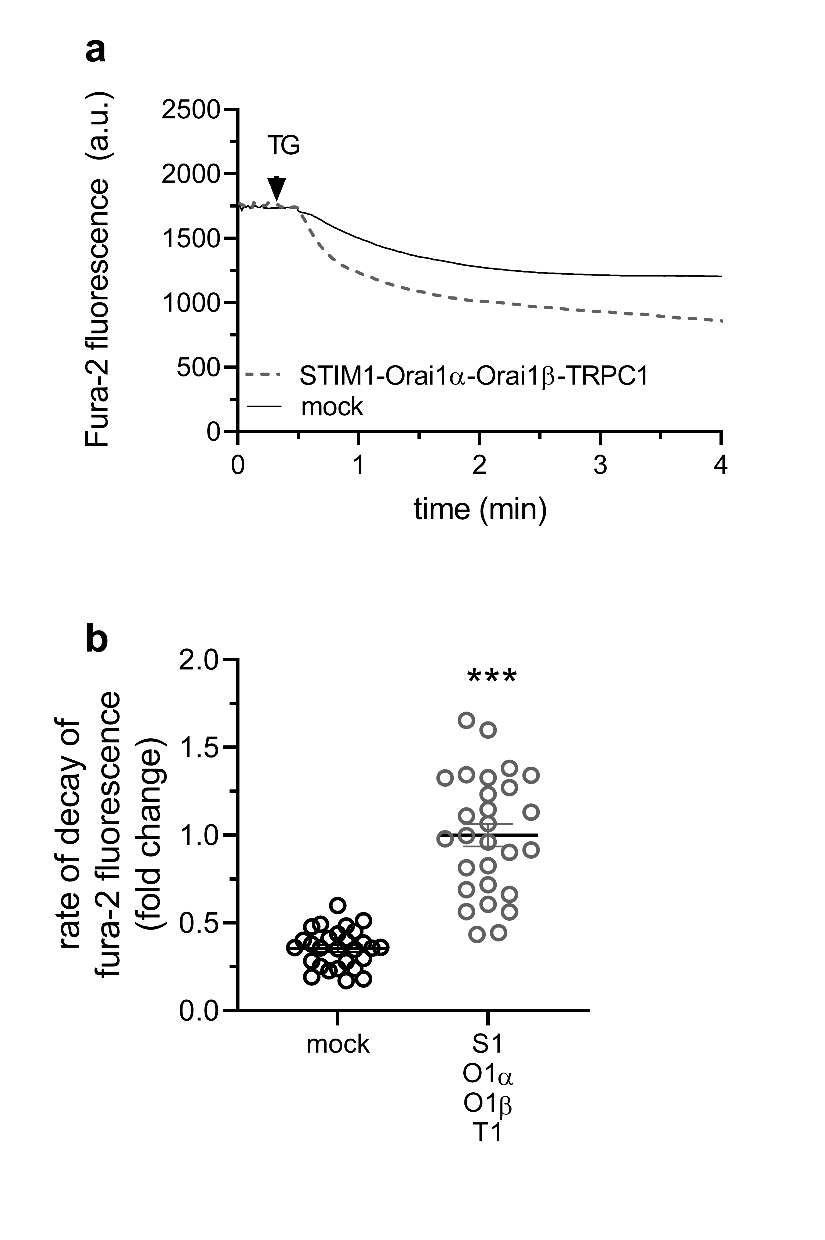
**

**Figure S7. Mn^2+^ influx in HeLa cells expressing STIM1, Orai1α, Orai1β and TRPC1.**

**a** Representative responses to 2 µM TG in HeLa cells co-transfected with STIM1, Orai1α, Orai1β and TRPC1 or mock transfected, as described. Cells were superfused with HBSS containing 0.5 mM Mn^2+^ and 1 mM Ca^2+^ and stimulated with 2 µM TG (indicated by arrow). Fura-2 fluorescence was measured at an excitation wavelength of 360 nm, the isoemissive wavelength. Representative traces were chosen to represent the datasets. **b** Quantification of the rate of decay of fura-2 fluorescence under the different experimental conditions (from left to right, n=28; n-values correspond to individual cells). Scatter plots are represented as mean ± SEM and were statistically analyzed using the Mann–Whitney U test. ****p*< 0.001 as compared to mock transfected HeLa cells.

**
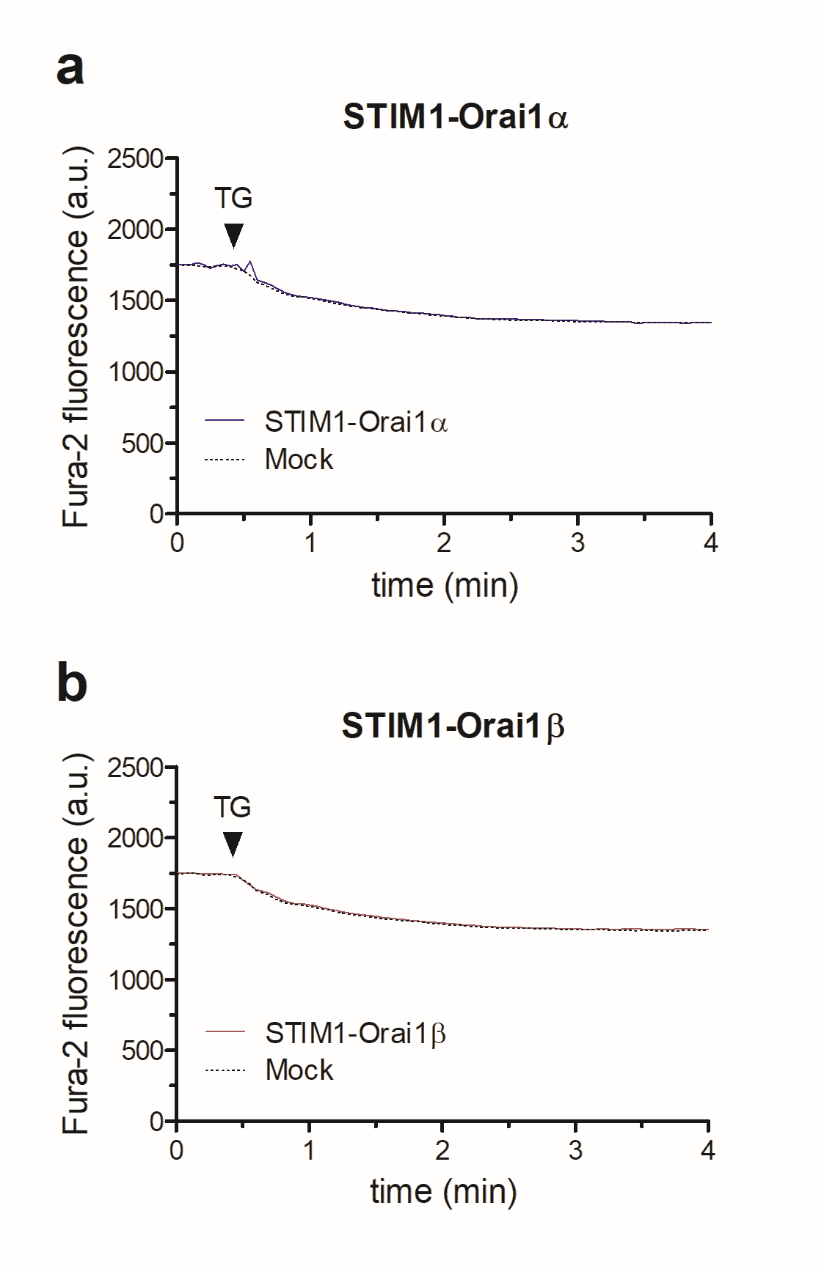
**

**Figure S8. Determination of Mn^2+^ influx in HeLa cells expressing STIM1 and Orai1α or STIM1 and Orai1β.**

Representative responses to 2 µM TG in HeLa cells co-transfected with STIM1 and Orai1α **(a)** or STIM1 and Orai1β **(b)**, as described. Cells were superfused with HBSS containing 0.5 mM Mn^2+^ and 1 mM Ca^2+^ and stimulated with 2 µM TG (indicated by arrow). Fura-2 fluorescence was measured at an excitation wavelength of 360 nm, the isoemissive wavelength. Traces are representative of 3 independent experiments (n=28-34; n-values correspond to individual cells).

**
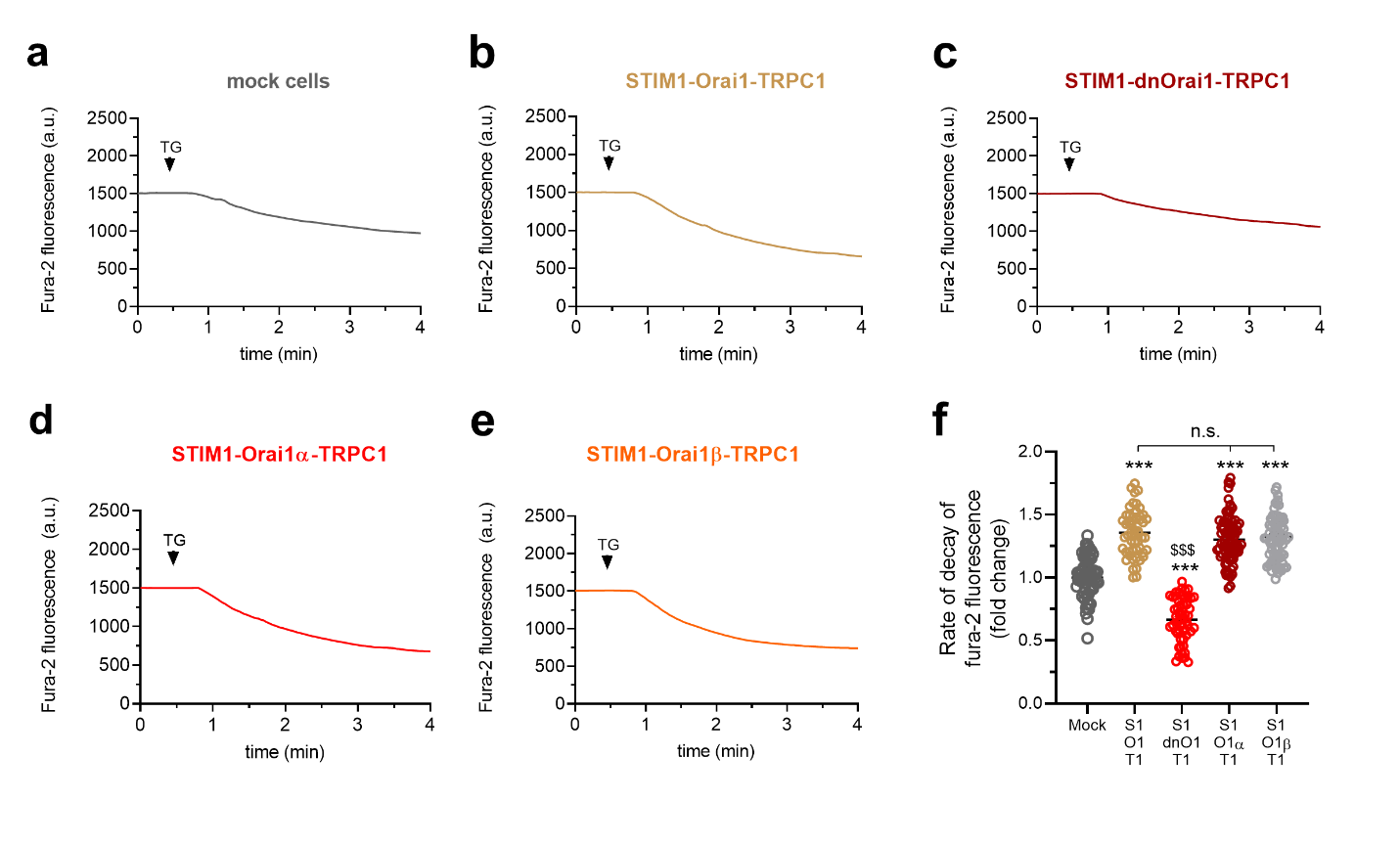
**

**Figure S9. Orai1α and Oraiβ modulate Mn^2+^ influx through TRPC1 in HEK293 cells.**

**a-e** Representative responses to TG in HEK293 cells co-transfected with empty vectors (mock cells; **a**), or expression plasmids for STIM1, TRPC1 and either EYFP-Orai1 (**b**), the dominant negative Orai1 mutant (**c**), Orai1α-EGFP (**d**) or Orai1β-EGFP (**e**), as described. Cells were superfused with HBSS containing 0.5 mM Mn^2+^ and 1 mM Ca^2+^ and stimulated with 2 µM TG (indicated by arrow). Fura-2 fluorescence was measured at an excitation wavelength of 360 nm, the isoemissive wavelength. Representative traces were chosen to represent the datasets. **f** Quantification of the rate of decay of fura-2 fluorescence under the different experimental conditions (from left to right, n=65, 53, 53, 78 and 70; n-values correspond to individual cells). Scatter plots are represented as mean ± SEM and were statistically analyzed using Kruskal–Wallis test with multiple comparisons (Dunn´s test). ****p* < 0.001 as compared to mock cells. ^$$$^*p* < 0.001 as compared to cells expressing STIM1, Orai1 and TRPC1.
